# Olfaction and Executive Cognitive Performance: A Systematic Review

**DOI:** 10.3389/fpsyg.2022.871391

**Published:** 2022-05-09

**Authors:** Vasudeva Murthy Challakere Ramaswamy, Peter William Schofield

**Affiliations:** ^1^School of Medicine and Public Health, Faculty of Health and Medicine, University of Newcastle, Callaghan, NSW, Australia; ^2^Neuropsychiatry Service, Hunter New England Mental Health, New Lambton, NSW, Australia

**Keywords:** olfaction, executive functioning, cognition, trauma, brain injury, review

## Abstract

Objective tests of olfaction are widely available to aid in the assessment of olfaction. Their clearest role is in the characterization of olfactory changes, either reported by or suspected in a patient. There is a rapidly growing literature concerned with the association of olfactory changes with certain neuropsychiatric conditions and the use of olfactory testing to supplement conventional assessments in clinical and research practice is evolving. Neural pathways important for olfactory processing overlap extensively with pathways important for cognitive functioning, and especially those important for executive functioning, many of which are concentrated in the frontal lobes. Previous work has identified associations between performance on certain olfactory tests (most frequently olfactory identification) and executive functioning and behavioral measures (e.g. of impulsivity). More recently, similar associations have also been identified in non-clinical samples, raising new questions as to the utility of olfactory test scores as proxy measures for non-olfactory phenomena. In this systemic review, we sought to identify studies, both clinical and non-clinical, that investigated the associations of olfaction with performance on tasks sensitive to frontal lobe functioning. Our search criteria led to the identification of 70 studies published in English. We examined in detail and tabulated the data from these studies, highlighted each study's key findings, and critically evaluated these studies. We use the results of this review to reflect on some of the current and future challenges concerning the use of olfactory testing in clinical neuropsychiatric practice and research and speculate on the potential benefits of administering phonemic fluency in combination with olfactory testing to enhance its predictive value.

## Introduction

Olfaction can be objectively assessed using standardized instruments that deliver an odor that the subject is required to detect or identify. The latter usually employs a forced-choice paradigm such that, typically, four options are given and the subject reports which of the four most closely corresponds to the odor (Hummel et al., 1997). Standardized, simple, and generally inexpensive objective olfactory tests are widely available for the clinical evaluation of patients and they have their clearest role in detecting, quantifying and monitoring acute olfactory loss such as might result from viral illness or head injury (Doty, 2007).

In addition, there has been growing interest in the potential of olfactory tests to supplement other clinical data collected in patients with a variety of neuropsychiatric syndromes often, but not always, in a research context. Thus, in large cohort studies of older individuals, lower performance on olfactory testing has been shown to represent a risk factor for subsequent cognitive decline and dementia (Devanand et al., 2015) and olfactory testing can aid in the differentiation of Parkinson's disease from other parkinsonian syndromes (Fullard et al., 2017).

The olfactory system comprises peripheral and central components. The central components are found in limbic and paralimbic structures which are situated in the anterior of the brain with a major cortical component located in the orbitofrontal cortex (Shipley and Ennis, 1996). Damage to the orbitofrontal cortex may result in changes in behavior while having little impact on performance on conventional neuropsychological tests (Jonker et al., 2015) and olfactory testing has been promoted as a possible objective probe for orbitofrontal dysfunction (Savic et al., 1997). Functional imaging studies demonstrate olfactory pathways linking cortical (cingulate, insula, frontal pole, hippocampus) and deep nuclei structures (amygdala), suggesting that olfactory performance might also be impacted by damage to parts of the frontal lobe lying dorsal and lateral to the orbitofrontal cortex (Arnold et al., 2020). Given that such structures contribute to performance on a range of “frontal lobe” or executive functioning (i.e., neuropsychological) tasks, it is not surprising that some studies have shown an association of olfactory performance with tests known to be sensitive to frontal lobe damage or dysfunction and the potential utility of olfactory testing as a quick and “simple” screen for cognitive impairment has been raised by a number of investigators. However, associations between olfactory and cognitive performance are not only limited to pathological populations but have also been found in non-pathological ones potentially complicating our understanding of the significance and clinical relevance of the former findings (i.e., in clinical populations) (Plailly et al., 2007). As far as we are aware, no systematic review has to date examined studies across the full spectrum (i.e., both in clinical and normal control populations) with respect to the association of olfactory and executive/”frontal lobe” tests, and we thought that by doing so, we might also usefully add to the ongoing discussions in the literature regarding the clinical value of olfactory testing in neuropsychiatric populations.

## Methods and Materials

This systematic review was conducted by extracting data from the identified studies, including, (1) study objective; (2) study design; (3) characteristics of study participants; (4) olfactory testing paradigms; (5) the presence of trauma, neurodegenerative disease, or psychiatric disease; (6) results of the study, and (7) key implications.

The search was conducted on July 7^th^ 2019 and a further limited search was done on March 27^th^ 2022. For each search, this systematic review was conducted in three stages. In stage one, a clear search strategy was developed to retrieve the relevant articles through different search engines. PubMed/MEDLINE, Scopus, EMBASE, Cochrane Library, Web of Science, PsycINFO, and Google Scholar were the search engines of choice, and controlled vocabularies were included when available (i.e., MeSH terms). Keywords were searched in the title/abstract fields. Keywords and combinations of TBI, brain hemorrhage, intracranial hemorrhage, brain edema, brain injury, brain damage, head injury, penetrating head injury, concussion, brain concussion, cerebral concussion, craniocerebral trauma, diffuse axonal injury, post-traumatic stress disorder (PTSD), olfaction, OD, sense of smell, olfactory impairment, neuropsychological, behavior, disinhibition, executive dysfunction, frontal lobe dysfunction, impulsivity, aggression, substance abuse, violence, brain tumors, frontal lobe tumors, meningioma, brain neoplasm, stroke, cerebrovascular disorder, cerebral palsy, cerebrovascular disorder, ruptured aneurysm, cerebral artery, degenerative diseases, dementia, AD, PD, Huntington's disease, frontotemporal dementia, multi-infarct dementia, personality disorders, impulsivity, aggression or addiction, substance abuse, drug addiction, violence, mood disorder, mania, and depression. No limitations were applied to the search; all languages, dates of publication (from the inception of the database to current), geographic areas, and ages of study participants were included. Animal and autopsy studies were excluded. Duplicate articles were removed.

In stage two, two investigators independently reviewed the titles and abstracts of articles to assess their eligibility for inclusion in this systematic review. Articles were regarded as relevant and warranted inclusion in this review if they were human studies, used validated olfactory testing methods, and examined for associations between olfaction and “frontal lobe” or executive functioning tasks.

### Data Extraction

**Figure 1:**
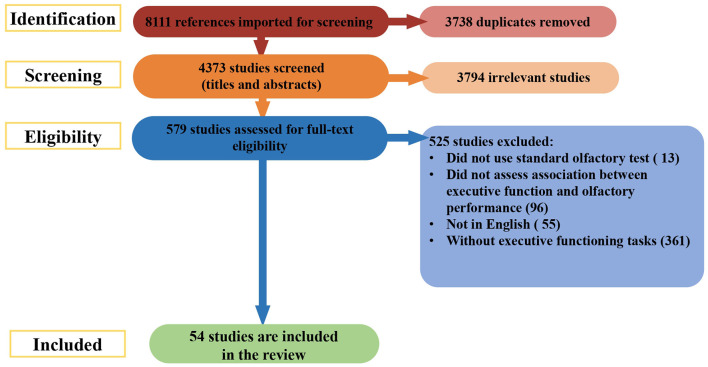
Prisma flow chart.

During stage 3, the titles and abstracts of articles were reviewed to determine their eligibility for inclusion in this systematic review. If there was uncertainty about whether a study should be included based on the title and abstract review, the full article was retrieved (see Figure 1 for article exclusion results).

The search of databases returned a total of 8,111 studies. During the initial stage, 3,738 duplicates were removed. The remaining 4,373 abstracts were screened for eligibility. Of these, 3,794 articles were excluded. The full texts of 579 records were reviewed for relevance; 525 were culled for the reasons listed in Figure 1. Fifty-four studies were included from this search.

**Figure 2:**
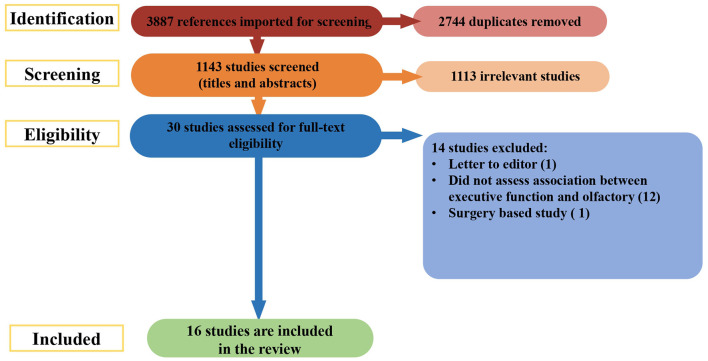
Prisma flow chart.

A further search was done on March 27^th^ 2022, covering the period from the initial search to the present. The search of databases returned a total of 3,887 studies. During the initial stage, 2,744 duplicates were removed. The remaining 1,143 abstracts were screened for eligibility. Of those, 1113 articles were excluded. The full texts of 30 records were reviewed for relevance; 14 were culled for the reasons listed in Figure 2. The remaining 16 new studies were included, making a final count of 70.

### Study Quality

The quality of the studies included in the systematic review was assessed with items adapted from the National Institutes of Health quality assessment tool for observational cohort and cross-sectional studies (Ma et al., 2020). We evaluated if: (a) the research question/objective of studying the correlation between olfactory capacity and neuropsychological performance was defined; (b) the study population was clearly defined (population demographics, location, and time period); (c) there was a >50% participation rate among participants recruited for entering the study; (d) all participants were recruited from the same/similar populations in the same time period and using the same inclusion/exclusion criteria (beside differences between patients and healthy groups); (e) the authors described the sample size estimation/study power and effect size estimates; (f) the exposure measures (independent variables) were clearly defined (e.g., reliable diagnostic tools); (g) the outcome measures (dependent variables) were clearly defined (e.g., reliable and validated instruments); (h) <20% of participants were excluded during the study; (i) critical confounding variables were taken into account. We added two additional items to assess if: (j) the correlation analyses were performed within all the groups included in a specific study (e.g., disease group and control/healthy group); and (k) the reporting of statistical results was adequate, with the description of exact *p*-values and test values for significant and non-significant results for the studies where measures of statistical significance were provided.

## Results

### Summary of the Studies

Supplementary Table 1 provides an overview of the study characteristics and main outcomes of the 70 studies. Most studies were cross-sectional comparative, with case and control populations (*n* = 34). Fewer studies were of cross-sectional design (*n* = 23), cohort prospective cohort (*n* = 11) and two studies were conducted retrospectively. Most studies were conducted in the United States (*n* = 20). Of the remainder, nine studies were conducted in Australia, four each in China and in the Republic of Korea, three each in Italy, Canada, Germany, Japan, Spain, Norway, Austria, two each in the United Kingdom and one in each in Croatia, the Netherlands, Czech Republic, Turkey, Chile, Belgium, France, and Sweden.

Studies included on average 141.43 ± 582. 69 (mean ± standard deviation) individuals per group (range 8–6,783 individuals) with a mean age of 54.97 ± 8.11 years (range 8–82 years), with 50.66 ± 24.74% of male participants (range 0–100%), and a mean education of 12.34 ± 1.57 years (range 5.52–17.5 years).

Supplementary Table 1 describes in detail the statistically significant and non-significant associations between olfactory performance and tests/questionnaires addressing frontal lobe function. Twenty-seven studies looked at neurodegenerative diseases and 15 at psychiatric conditions. There were 20 studies among healthy populations and eight studies among a population with TBI.

The most commonly used olfactory tests were the UPSIT (*n* = 25) and SS (*n* = 20), nine studies used the Brief-Smell Identification Test (B-SIT), three studies used Cross-Cultural Smell Identification Test, two studies used Pocket Smell Test Score Odor Stick Identification Test for Japanese and the Scandinavian Odor-Identification Test, the other olfactory performance tests were used only once per study. All the studies measured olfactory identification (*n* = 70), while fewer publications addressed olfactory discrimination (*n* = 12) and olfactory detection (*n* = 10) (Supplementary Table 1).

The most commonly used frontal lobe tests/questionnaires were the verbal fluency tests (*n*=26). Among 26 studies that used verbal fluency, category/phonemic/letter fluency (*n* = 14) was used most followed by the Controlled Oral Word Association Test (COWAT) (*n* = 9) and the Animal Fluency Task (AFT, *n* = 8). In five studies more than one type of verbal fluency test was used.

The other tests frontal lobe tests/questionnaires which were used were: the Trail Making Test-B (TMT-B, *n* = 22), the Wisconsin Card Sort Test (WCST, *n* = 16), the Rey-Osterrieth Complex Figure Test (ROCFT) copy (*n* = 10), the Stroop Color and Word Test (SCWT, *n* = 12), WAIS-Digit span (*n* = 19), the Iowa Gambling Test (IGT, *n* = 5), Digit Substitution Test (*n* = 4), CWIT (*n* = 3), the Stop-Signal task (SST, *n* = 3), the Tower of London (*n* = 3), the Frontal Assessment Battery (FAB, *n* = 3), the Barratt Impulsivity Scale (*n* = 2), the Delayed Alternation Task (DAT, *n* = 2) and the Information Sampling Task (IST, *n* = 2). The executive function domains associated with each test are represented in Supplementary Table 1.

### Correlation Between Olfactory Function and Frontal Lobe Measures

This section reports on the findings of correlations between tests that have been described to engage the frontal lobe and some degree of OD (detection, discrimination, and identification). Among the studies reviewed, there was heterogeneity with respect to the olfactory and neuropsychological domains measured; thus, we specify the precise measures in each instance. Statistically significant and non-significant correlations are reported. The studies also used different statistical tests (e.g., Pearson/Spearman's correlation and linear/logistic regression). Some authors controlled for putative confounding variables (e.g., age, gender, education, and smoking) while others did not. Supplementary Table 1 summarizes this information.

#### Impulsivity

Two studies found an inverse association between impulsivity (Eysenck's Impulsivity and Barratt Impulsivity Scale) and olfactory performance: in war veterans suffering from PTSD (Dileo et al., 2008) and among healthy volunteers (Herman et al., 2018). In two additional studies using the BSIT odor identification, no significant correlations with impulsivity (Barratt Impulsivity Scale) scores among former American football players (Alosco et al., 2017), or with the IST in an OCD study population (Bersani et al., 2013), were found. Another study using UPSIT in the AD study sample reported no significant correlations with the Balloon Analog Risk Task (Ward et al., 2016).

#### Processing Speed

In a sample with multiple sclerosis patients and control participants, a positive association was found between processing speed (Symbol Digit Modalities Test) and UPSIT olfactory identification (Carotenuto et al., 2019). BSIT identification was also positively associated Digit Symbol Coding Test among former American football players (Alosco et al., 2017).

#### Inhibitory Control

Positive associations between SCWT and olfactory performance were found among individuals with PD (Hanoglu et al., 2014), AD (Lehrner et al., 2009; Park et al., 2018), children and teenagers with early-onset psychosis (Corcoran et al., 2005), patients with schizophrenia (Purdon, 1998), late life depression (Chen et al., 2018), and healthy individuals (Fagundo et al., 2015). In a study with late life depression participants, olfactory performance was associated with Stroop A but not with Stroop Interference Effect (Chen et al., 2018). However, our searches also identified studies among those with PD (Morley et al., 2011; Yoo et al., 2019), AD (Lian et al., 2019), TBI (Langdon et al., 2021), and community-dwelling adults (Goette et al., 2017), in which significant associations between olfactory identification and SCWT was not found.

Two studies found a significant association between the SST scores and olfactory performance (Bersani et al., 2013; Herman et al., 2018). The SS odor detection threshold was positively correlated with SST and SCWT among healthy participants (Fagundo et al., 2015; Herman et al., 2018). Both odor sensitivity and discrimination statistically significantly predicted the inhibitory control SST (Herman et al., 2018), and the prolonged response inhibition Stop-Signal reaction time strongly correlated with the BSIT score. Similarly, Maurage and colleagues did not find statistically significant associations between SS olfactory identification, discrimination, and detection (also retronasal identification) and performance of SST in alcohol-dependent and healthy participants (Maurage et al., 2011).

A study in children with TBI reported no significant correlations between the “walk don't walk” task and color word interference with olfactory identification (Bakker et al., 2017). The Go/No-go task conflict score was negatively correlated with olfactory identification in healthy subjects (Alberta Smell Test; left nostril) (Spinella, 2002). In a study with TBI participants, the olfactory performance didn't predict the observed disinhibition or informant-reported disinhibition (Osborne-Crowley and McDonald, 2016).

#### Verbal Fluency

Significant positive associations between measures of olfaction and performance on category and letter fluency tests have generally been found in healthy study populations (Larsson et al., 2004; Hedner et al., 2010; Goette et al., 2017; Choi et al., 2018; Yahiaoui-Doktor et al., 2019), in mixed clinical and control samples (Parrao et al., 2012; Vyhnalek et al., 2015; Carotenuto et al., 2019), in clinical populations with TBI (Sigurdardottir et al., 2010, 2016; De Guise et al., 2015; Bakker et al., 2017), in older individuals or those with cognitive impairment or AD (Westervelt et al., 2005; Devanand et al., 2010; Tkalčić et al., 2011; Choi et al., 2018; Park et al., 2018; Churnin et al., 2019; Turana et al., 2020), old veterans (Freimer et al., 2019) and early psychosis (Corcoran et al., 2005). However, verbal fluency was not significantly associated with olfactory measures in patients with PD with or without controls (Parrao et al., 2012; Hanoglu et al., 2014) or in a sample of amyotrophic lateral sclerosis (ALS) patients and healthy participants (Parrao et al., 2012), in early frontal AD pathology (Lehrner et al., 2009) or TBI participants (Callahan and Hinkebein, 1999) or individuals at risk for psychosis and patients with schizophrenia (Takahashi et al., 2018). Similar significant associations were reported in several studies, fluency measures were compared between groups defined by the presence or absence of olfactory impairment. These studies, involving patients with past TBI (Callahan and Hinkebein, 1999; Langdon et al., 2021), schizophrenia (Takahashi et al., 2018) and PD (Yoo et al., 2019, 2020) found no group-specific differences in fluency-test performance. Overall, most studies that included the measures showed a positive association between verbal fluency performance and olfactory identification capacity.

#### Working Memory

An association between digit span performance with olfactory performance was found in several studies (Westervelt et al., 2005; Bahar-Fuchs et al., 2010; Hedner et al., 2010; Morley et al., 2011; Segalàs et al., 2011; De Guise et al., 2015; Alosco et al., 2017) but not in others (Brewer et al., 1996; Saoud et al., 1998; Callahan and Hinkebein, 1999; Vasterling et al., 2003; Lehrner et al., 2009; Parrao et al., 2012; Vyhnalek et al., 2015; Freimer et al., 2019; Lecuyer Giguère et al., 2019; Lian et al., 2019; Yoo et al., 2019, 2020; Langdon et al., 2021).

#### Mental Flexibility

Eight studies found significant positive associations between (better) WCST and olfactory performance: in patients with PD (Parrao et al., 2012; Yoshii et al., 2019), schizophrenia (Brewer et al., 1996; Saoud et al., 1998), TBI (Callahan and Hinkebein, 1999) alcohol dependence (Rupp et al., 2006), as well as healthy participants as part of an assessment of sleep deprivation (Killgore et al., 2010) and community-dwelling adults (Goette et al., 2017). In several studies no association between olfactory scores and WCST was found: in a sample of schizophrenic patients (Seidman et al., 1997), healthy individuals with hyposmia and normosmia (Fagundo et al., 2015), young people (Gellrich et al., 2021), and individuals with TBI (Langdon et al., 2021).

Eleven studies found a significant association between TMT-B and olfactory performance. This included studies of patients with TBI (Callahan and Hinkebein, 1999; De Guise et al., 2015), early-onset psychosis (Corcoran et al., 2005), dementia (Westervelt et al., 2005), American football players (Alosco et al., 2017), old veterans (Freimer et al., 2019), community-dwelling adults (Goette et al., 2017), subtypes of mild cognitive impairment (Lehrner et al., 2009), AD (Tahmasebi et al., 2020) and healthy subjects (Yahiaoui-Doktor et al., 2019; Kose et al., 2021).

However, nine studies reported non-significant correlations between olfactory performance and TMT-B. Study populations in these “negative” studies included mixed samples of healthy controls and patients with PD (Parrao et al., 2012), cognitive impairment (Vyhnalek et al., 2015), amyotrophic lateral sclerosis (Pilotto et al., 2016), TBI (Bakker et al., 2017; Lecuyer Giguère et al., 2019; Langdon et al., 2021), AD (Wang et al., 2021), late life depression (Chen et al., 2018) and bipolar disorder (Hardy et al., 2012). In healthy participants as part of an assessment of sleep deprivation, SIT and the color trailing test did not correlate significantly (Killgore and McBride, 2006). In a study of individuals with a history of TBI, some of whom were administered the B-SIT and others the UPSIT, associations were found between BSIT, but not the UPSIT, with TMT (Sigurdardottir et al., 2016).

#### Decision-Making

Studies that examined the association of olfaction with the IGT were also mixed Significant associations were found in healthy study participants (Bettison et al., 2013) and in a study comparing Alzheimer's disease, mild cognitive impairment, and healthy older adults, (Ward et al., 2016) but non-significant associations were found in other studies (Fagundo et al., 2015; Goette et al., 2017).

#### Visuospatial Processing

Several studies have examined the association of olfactory measures with the ROCFT copy test. The majority (Westervelt et al., 2005; Bahar-Fuchs et al., 2010; Parrao et al., 2012; Kjelvik et al., 2014; Vyhnalek et al., 2015; Pilotto et al., 2016; Alosco et al., 2017; Goette et al., 2017; Park et al., 2018; Lian et al., 2019; Mertens et al., 2019; Yoo et al., 2019, 2020; Wang et al., 2021) found no significant association, while three studies did find such an association (Pilotto et al., 2016; Chen et al., 2018; Lian et al., 2019).

Similarly, four studies (Seidman et al., 1997; Corcoran et al., 2005; Hanoglu et al., 2014; Langdon et al., 2021) did not find an association of olfactory scores with the Block Design Test, although a study in healthy participants did (Larsson et al., 2004).

#### Planning

In a study of individuals with PD, significant correlations of olfactory measures with the Tower of London test were noted (Morley et al., 2011). Similarly, significant correlations were found with the Tower of Hanoi and olfactory measures (Larsson et al., 2004). However, in studies of individuals with PD and healthy participants (Parrao et al., 2012) and study with schizophrenic patients (Takahashi et al., 2018) no significant correlations of olfactory measures with the Tower of London test were noted.

#### General Executive Function

The Frontal Assessment Battery (FAB) is an instrument comprising of multiple tests, the scores of which are summed up to provide a measure of frontal lobe functioning. The FAB and olfactory function were significantly different between amyotrophic lateral sclerosis - frontotemporal dementia (ALS-FTD) spectrum patients and controls (*p* < 0.001) (Pilotto et al., 2016). The FAB was not correlated with performance on odor tests (detection, identification, and discrimination) in patients with dementia and control participants; however, this was a very small study (total *n* = 20) (Orasji et al., 2016). Another study did not find a significant statistical association in patients with dementia using the Delis–Kaplan Executive Function System (DKEFS) test and the UPSIT identification score (Pardini et al., 2009). Within a population of elderly adults with and without smell impairment, smell identification function (Pocket Smell Test Score) was positively associated with executive functioning using the Digit Symbol Substitution Test (Choi et al., 2018) and with multiple sclerosis participants significant correlation were found with UPSIT and Symbol Digit Modalities Test (Carotenuto et al., 2019). Additionally, in another study using the Pocket Smell Test with a similar sample, the identification score was positively associated with Digit Symbol Substitution Test (Churnin et al., 2019). Another study with healthy participants also found a positive correlation between identification scores (Scandinavian Odor-Identification Test) and the Letter-Digit Substitution test (Larsson et al., 2004).

#### Other Measures of Executive Function

In a study that included individuals with early-onset Alzheimer's disease and those with mild cognitive impairment, olfactory identification correlated with executive functions of Cambridge Mental Disorders of the Elderly Examination within the early-onset AD group only (Velayudhan et al., 2019). In a sample of patients with PD, the Visual Executive Test, a subset of the Montreal Cognitive Assessment, was a significant predictor of UPSIT scores (Mertens et al., 2019).

### Summary of Significant vs. Non-significant Results by Executive Function Domain, Olfactory Domain, and Disease Type

We assessed the results by medical condition, olfactory test, and frontal lobe test. Fifty three articles reported at least one significant association between olfactory performance and frontal lobe testing, while 17 articles reported only non-significant associations. Most studies did not present analytical data when results were not statistically significant (neither test value nor the exact *p*-value; more information in the quality assessment Section). Therefore, unless explicitly mentioned, we will be conservative and assume that there was no significant correlation.

When stratifying by medical condition, significant associations were found in fifty three studies. Five trauma studies found significant associations. There were 22 studies with significant associations among neurodegeneration patients, 11 among psychiatric patients, and 15 among healthy patients. Only non-significant associations were found in 17 studies: three studies were among TBI studies, five studies were among neurodegeneration patients, four studies were psychiatric patients and five were in healthy population. In TBI patients, the olfactory identification score was positively correlated with verbal fluency and working memory abilities and negatively correlated with mental flexibility. In neurodegenerative diseases, the identification score was positively correlated with inhibitory control, mental flexibility and verbal fluency. For patients with psychotic disorders, the identification score was positively correlated with inhibitory control, verbal fluency, attention and mental flexibility. In healthy samples, the identification score was negatively correlated with planning abilities and positively correlated with verbal fluency, the discrimination score was negatively correlated with impulsivity and inhibitory control and positively associated with verbal fluency, and the detection score was positively associated with inhibitory control and processing speed. However, we need to carefully consider these conclusions because some studies performed a joined analysis of healthy and clinical samples (more information in the quality assessment Section).

When stratifying by the olfactory test, the majority of significant associations were found for studies using the UPSIT (*n* = 19/53), SS (*n* = 17/53), BSIT (*n* = 6/53), Pocket Smell Test (*n* = 2/53), Scandinavian Odor-Identification Test (*n* = 2/53), Cross-cultural smell identification test (*n*=2/53), The Odor Memory Test (*n* = 1/53), Alberta Smell Test(n=1/53), Odor Stick Identification Test for Japanese (*n* =1/53), Connecticut Chemosensory Clinical Research Centre Test (*n* =1/53), Sentosphère olfactory identification test (n=1/53), Motol Hospital Smell Identification Test (*n* =1/53) and Olfactory Detection Threshold (*n* =1/53) among other tests used only once per study (see Supplementary Table 1). By stratifying based on the subset of olfactory tests among significant studies, fifty three studies did report significant associations for olfactory identification tests, 10 studies for olfactory discrimination, and eight studies for olfactory detection.

Among the 26 studies which used verbal fluency studies, 21 studies found at least one significant association. Among them COWAT (*n* = 5/9), Animal Naming test (*n* = 7/8) and category/phonemic/letter fluency (*n* = 13/14). In five studies more than one types of verbal fluency test was used, hence the data is doubly represented.

When stratifying by the other frontal lobe test, studies that reported at least one significant associations used the TMT-B (*n* = 16/22), WCST (*n* = 12/16), ROCFT (*n* = 10/13), SCWT (*n* = 10/12), WAIS-Digit (*n* = 12/19), IGT (*n* = 5/5), Digit Substitution test (*n* = 4/4), Tower of London (*n* = 3/3), Stop-Signal task (*n* = 3/3), Information Sampling Task (*n* = 2/2), COWAT (*n* = 5/9), CWIT (*n* = 1/3), Barratt Impulsivity Scale (*n* = 2/2), The Balloon Analogue Risk Task (*n* = 1/1), Walk Don't Walk (*n* = 1/1), Maze test (*n* = 1/1), Tower of Hanoi (*n* = 1/1) and Eysenck's Impulsivity Questionnaire (*n* = 1/1).

### Studies Quality Assessment

The assessment of the quality of the studies is represented in Supplementary Table 2. All the studies adequately defined the aims of the study. All the studies provided an adequate definition of the study population(s) (except Spinella, 2002), but some failed to report appropriate exclusion criteria Callahan and Hinkebein, 1999; Spinella, 2002; Morley et al., 2011; Tkalčić et al., 2011; Hardy et al., 2012; Osborne-Crowley and McDonald, 2016; Goette et al., 2017; Blanco et al., 2019; Mertens et al., 2019; Gellrich et al., 2021; Kose et al., 2021). Almost all studies provided an indication of the participation rate (except Callahan and Hinkebein, 1999; Vasterling et al., 2000; Spinella, 2002; Maurage et al., 2011; Morley et al., 2011). Most of the studies did not report a justification of sample size or calculation of power and estimation of effect sizes (except Vasterling et al., 2003; Sigurdardottir et al., 2010; De Guise et al., 2015; Vyhnalek et al., 2015; Ward et al., 2016; Cha et al., 2021). Regarding blinding of outcome assessors, only four studies described that the assessors or participants were blinded (Seidman et al., 1997; Purdon, 1998; Killgore et al., 2010; Park et al., 2018). Moreover, few of the studies did not report how many participants were excluded during the study (except Seidman et al., 1997; Purdon, 1998; Larsson et al., 2004; Westervelt et al., 2005; Rupp et al., 2006; Bahar-Fuchs et al., 2010; Hedner et al., 2010; Sigurdardottir et al., 2010; Morley et al., 2011; Hardy et al., 2012; Bettison et al., 2013; Devanand et al., 2015; Pilotto et al., 2016; Ward et al., 2016; Alosco et al., 2017; Bakker et al., 2017; Takahashi et al., 2018; Yahiaoui-Doktor et al., 2019; Turana et al., 2020; Cha et al., 2021; Gellrich et al., 2021) or reported an exclusion rate higher than 20% (Devanand et al., 2010; Churnin et al., 2019). More than half of the studies controlled for potential confounding variables (e.g., age, gender, education, and smoking) and performed statistical analysis within each study group (e.g., patients and control group). Finally, most of the studies reported p-values for significant results with adequate statistical information when describing the results (except Purdon, 1998), where information on the statistical method used was not available. The *p*-values for non-significant results were not reported for some of the studies (Brewer et al., 1996; Seidman et al., 1997; Saoud et al., 1998; Barnett et al., 1999; Callahan and Hinkebein, 1999; Killgore and McBride, 2006; Rupp et al., 2006; Hedner et al., 2010; Maurage et al., 2011; Tkalčić et al., 2011; Hardy et al., 2012; Parrao et al., 2012; Bersani et al., 2013; Bettison et al., 2013; Vyhnalek et al., 2015; Orasji et al., 2016; Pilotto et al., 2016; Alosco et al., 2017; Herman et al., 2018; Velayudhan et al., 2019).

## Discussion

In this study, substantial evidence of associations between olfactory performance (in almost all instances on an olfactory identification test) and tests of executive function, and impulsivity was found, albeit not consistently. These included associations between olfaction and working memory, mental flexibility, and visuospatial processing. The most significant associations with olfactory identification capacity and inhibitory control tasks performance (mainly SCWT) and verbal fluency (letter and category fluency) were found in neurodegenerative, multiple sclerosis, psychiatric (psychosis and schizophrenia), TBI, and healthy populations. The findings of associations between olfactory performance and impulsivity (negative) and inhibitory control (positive) in multiple studies with different population characteristics were perhaps most anticipated, given the central importance of the orbitofrontal lobe for both olfaction and behavioral regulation. Indeed, to the extent that olfactory testing has been recommended as an adjunct to cognitive testing it has often been as a probe of the orbitofrontal cortex (Savic et al., 1997).

The roster of pathological conditions featured in this systematic review reflects the fact that both olfactory changes and cognitive impairment frequently co-occur among individuals with those conditions. While there was unavoidably variation in the specific tests of executive/frontal lobe functioning, there was also a good deal of overlap across the studies, allowing for some meaningful comparisons to be made. We acknowledge that restricting our analyses to correlations, and our reliance on statistical significance in the appraisal of the correlations, could be regarded as important limitations. While it has become less favored to emphasize statistical significance, however, this approach served to mitigate otherwise potentially inflated contributions from small studies.

It is important to consider the multiple means by which the associations of olfaction and cognition we have identified might potentially arise. The first, simplest and most direct mechanism is the presence of pathology that damages a single brain region whose integrity is important both for olfactory and cognitive processes. One important such region is the OFC. The OFC corresponds to the principal cortical target of the primary olfactory cortex which plays a key role in odor processing (Gottfried and Zald, 2005), odor discrimination, identification, and memory (Martzke et al., 1997). Functional imaging studies demonstrate the substantial overlap between neural substrates for olfactory identification, discrimination and executive functioning, (Drevets, 2000; Zald and Pardo, 2000; Torregrossa et al., 2008; Han et al., 2019; Friedman and Robbins, 2022). OFC integrity is important for higher-level cognitive functions such as working memory, self-control, and decision making (Bechara et al., 2000; Wallis, 2007) and damage to the OFC is associated with impulsivity (Berlin et al., 2004; Spinella, 2004; Torregrossa et al., 2008) and disinhibited behavior (Schoenbaum et al., 2007). Head trauma has substantial potential for causing damage to the OFC, with consequences for olfaction and behavior (Schofield et al., 2014). However, post-traumatic olfactory dysfunction may also arise due to (peripheral) damage to the olfactory nerve without associated brain damage or cognitive sequelae following what would otherwise be regarded as mild TBI. Thus, while more common following severe TBI, the olfactory loss does not serve as a reliable index of past TBI severity (Sigurdardottir et al., 2010; Schofield et al., 2014).

A second possible mechanism of association, which might in part account for the findings in some individuals following TBI or those with neurodegenerative conditions characterized by diffuse pathology, is contemporaneous damage to both olfaction- and cognition-critical networks, even if these are topographically segregated. Among patients who sustain a TBI – leaving aside those in whom damage to OFC or adjacent structures clearly explains the olfaction/cognition association – some will have dual pathology, for example, olfactory nerve (i.e., a peripheral lesion to cause hyposmia/anosmia) together with cognitive sequelae due to contusions or other brain damage remote from olfactory pathways. As mentioned above, the heterogeneity of pathology following TBI likely accounts for the inconsistent relationships reported in the literature with respect to olfaction and cognition. By contrast, the pathology of Alzheimer's disease, and to a lesser extent Parkinson's disease, evolves in a rather stereotypical way (Braak et al., 2011). While the pathology of AD ultimately damages extensive subcortical and cortical regions, where the considerable topographical overlap of relevant pathways occurs, we suggest that multisite, non-overlapping contemporaneous pathology affecting, separately cognition and olfaction, may plausibly account for some of the associations found in large samples. AD and PD are associated with relatively early pathology in the olfactory bulb and in the case of PD especially, olfactory changes may arise before cognitive decline (Costanzo and Nathan, 1992; Ponsen et al., 2009; Fullard et al., 2017; Park et al., 2018). Study populations comprising individuals with a broader range of severity (greater variance of the measures under study) would be expected to yield stronger associations. In psychiatric populations, where structural pathology is for the most part lacking or not detectable, data from functional imaging studies can be informative. Thus, studies of individuals with schizophrenia demonstrate hypo-functioning in cerebral regions that harbor olfactory and cognitive networks, providing an explanation for the syndrome of associated deficits (Minzenberg et al., 2009; Kiparizoska and Ikuta, 2017).

A third possible mechanism of an association of olfactory performance with cognition is one in which specific cognitive deficits negatively impact the *process* of olfactory testing. Thus, deficits in semantic processing or naming may cause errors during olfactory identification trials that might not accurately reflect actual (essentially unmeasurable!) olfactory capacity (Olofsson et al., 2013). Similarly, inattentiveness or impulsivity, particularly for olfactory tests that take longer for the subject to complete, such as the 40 item UPSIT, might lead to “careless errors” thereby lowering olfactory test scores. We are unaware of published evidence in support of the latter hypothetical mechanism, but our experiences in testing a large cohort of impulsive offenders suggest this as a possibility (Butler et al., 2021).

A final mechanism is one in which sensory and cognitive test performance is intrinsically associated, leading to positive correlations of cognitive (“frontal lobe”) testing with olfactory performance in the absence of underlying pathology (Danthiir et al., 2001; Meyer et al., 2010; Dahmani et al., 2018; Gellrich et al., 2021). As noted in the present study, there is now substantial evidence for this phenomenon.

Before addressing the question of whether our results have any important implications for patient care or research, we need to acknowledge some further limitations of this work. As mentioned earlier, significant correlations can arise for a variety of reasons. Variability of the findings with respect to the associations of cognition with olfaction in this review will have been influenced by the spectrum of severity, clinical variability within the study populations and the nature of the measurements undertaken. The review was undertaken in two parts, reflecting an unavoidable interruption in the project due to COVID, and related health and travel considerations affecting the first author. The scope of the search did not deliberatively include memory, although several of the included studies did examine that construct. We do not believe, however, that this lack significantly diminishes the value of the work.

As mentioned earlier, a major use of simple clinic testing of olfaction is to detect olfactory impairment for which there are many recognized causes (Mullol et al., 2012) Some but not all of the studies included in this review sought, in their recruitment strategies, to exclude individuals who might have sustained olfactory loss due to sinus or other local nasal problems, or due to medications. Given that some individuals will be unaware of olfactory loss, there remains the possibility, even with careful historical screening, that poor olfactory test performance could be of long standing and/or on the basis of peripheral pathology and not therefore informative with respect to central nervous system pathology. The success or lack thereof of efforts to exclude such individuals in research studies will likely impact the psychometric properties of testing. Such consideration aside, the now widely demonstrated association of cognitive changes with olfactory test performance has led some investigators to suggest that olfactory testing might constitute a simple means of screening for cognitive impairment. How do our results bear on that suggestion?

There are very limited data on the *cross-sectional* psychometric properties of olfactory testing (i.e. as distinct from prediction of *subsequent* cognitive decline) in terms of sensitivity/specificity etc. for some cognitive outcome. In the Life-Adult study, which used the 12-item form of the Sniffin Sticks', a olfactory identification test, while highly significant univariate and multivariate associations of executive function tests (verbal fluency, Trail making test B/A) were identified, Receiver operating curve analyses showed very modest scores (AUC 0.55 for verbal fluency, 0.55 for TMT and corresponding sensitivity/specificity scores) leading the investigators to comment that “the ability of the smell test to discriminate between individuals with and without cognitive impairment was limited” (Yahiaoui-Doktor et al., 2019). A clear implication from that finding is that olfactory testing using that specific test is unlikely to provide a “simple” means of screening for cognitive impairment; testing cognition, perhaps in creative ways, is more direct and effective (Schofield et al., 2010). Similarly, for example, if the behavioral correlates of OFC damage were a matter of clinical concern, direct measures of the problematic behaviors, such as by administering an impulsivity questionnaire, should be considered in preference to olfactory testing, at least while major questions of interpretation remain unresolved. Such problems aside, however, we can report that in an ongoing study of impulsive violent men to whom we have been administering the 16-item “Sniffin Sticks,” the procedure has been extraordinarily well-accepted and is seen as a fun interlude in the course of other data collection (Butler et al., 2021).

With respect to the focus of this review on correlates of olfactory performance, we believe there is currently insufficient evidence to suggest that olfactory testing can reliably provide insights into cognitive/behavioral functioning, at the individual clinical level, that are not obtainable by other more conventional means. More studies to specifically explore the diagnostic/ evaluative properties of olfactory testing, compared head to head with other measures (such as phonemic fluency, or more sophisticated tests of reversal learning) would be interesting and valuable. In designing such studies, investigators would ideally bear in mind the resources and screening that could reasonably be employed by clinicians who might ultimately use olfactory testing, were it to be shown to have good sensitivity and specificity. For example, the inclusion in such studies of only patients who have undergone a formal rhinological assessment to exclude forms of local (i.e., peripheral pathology), while clearly laudable at one level, would constrain the generalizability of the findings (as it is hard to imagine that such preliminary assessments would always be done in clinical practice) and potentially exaggerate the favorable properties of testing (if applied in individuals not as well-screened for peripheral disease). Perhaps, in an ideal world, researchers could recruit enough study subjects to enable stratification according to the intensity of the initial evaluation of rhinological status (allowing for, in one strata, exclusion of those with olfactory problems possibly due to peripheral olfactory conditions). Numerous other methodological considerations arise. Which olfactory test(s) should be evaluated for the above recommended studies? Given the investments of effort and time that would be needed, including a range of olfactory tests, both long and well-validated as well as shorter, cheaper and, if possible, widely available tests would be advantageous. On balance, we suspect that even if such a program of research were to be undertaken, the clinical utility of olfactory testing, besides the scenarios we have already touched upon, will be limited.

There seems to be better evidence to support the value of olfactory testing as a potential predictor of cognitive decline, while recognizing that the lack of specificity of olfaction as “biomarker” for neurodegeneration does represent a limitation. In relation to this, however, there have been some interesting findings. Several studies have noted that olfactory impairment has greater salience – is a stronger predictor of decline – among individuals who carry the APOE 4 allele (Graves et al., 1999; Olofsson et al., 2020). One way to interpret this finding is that the presence of a known strong risk factor for AD (i.e., APOE ε4) increases the likelihood that olfactory impairment, when present, has a neurodegenerative (central not peripheral) basis. Other investigators, perhaps inspired by observations that discordance between subjective and informant report of dysfunction may predict cognitive decline (Tabert et al., 2002), have examined the significance of unawareness of olfactory loss, as determined objectively by testing (Tahmasebi et al., 2020). Against this background, we would like to highlight the impressive consistency within the non-normative populations (i.e., those defined by the various pathologies), of a significant association of olfaction (mostly olfactory identification) with verbal (phonemic) fluency. Of the many tests administered by researchers in the studies we reviewed, phonemic fluency is one of the easiest and most convenient to administer and score, and one for which there is abundant normative data. Importantly, the topographic affiliations of verbal fluency have become increasingly clear, with evidence to indicate that the left inferior frontal gyrus (IFG) is an important component of the relevant networks that contribute to its performance (Schlösser et al., 1998; Robinson et al., 2012). IFG lies adjacent to OFC with which it has strong connections (Du et al., 2020). In a recent study, that examined Dynamic Functional Connectivity (DFC) as a possible biomarker for early Alzheimer's disease, DFC variability in the right middle temporal gyrus and in the left IFG were significantly different between controls and patients with subjective cognitive impairment and amnestic mild cognitive impairment (Xue et al., 2021) suggesting left IFG as a region whose function may be highly informative with respect to AD risk. Accordingly, and by analogy with the interaction of APOE ε4 and olfaction described immediately above, we speculate that, if extremely brief screening for risk stratification were planned based upon the use of olfactory testing, the interaction of phonemic fluency performance with olfaction might be worth examining in relation to predicting decline. We are not aware of any studies that have specifically explored this possibility.

In conclusion, olfactory discrimination scores derived from simple olfactory testing are helpful in characterizing olfactory function but currently, we believe, add little to the detection of cognitive impairment syndromes. Although not the focus of this review, olfactory testing, in combination with other tests, may ultimately contribute to the quantification of risk for cognitive decline.

There are many neuropsychiatric conditions in which olfactory changes have been noted, consideration of which exceeded the scope of this review (Brewer et al., 2006). There is much more to be learned about olfaction, its relationship to other brain systems, and its importance for quality of life. The advent of Covid, which has given rise to a large cohort of individuals who sustained (mostly transient) olfactory loss, has invigorated clinical research into olfactory disorders, and the future looks rich with possibility.

## Data Availability Statement

The original contributions presented in the study are included in the article/Supplementary Material, further inquiries can be directed to the corresponding author.

## Author Contributions

VC and PS defined the objectives and assessed the relevance of abstracts and full-text articles. VC extracted the data, assessed the risk of bias in individual studies, analyzed and interpreted the results, and wrote the first draft of the manuscript. PS critically reviewed and edited the early drafts and contributed to writing the final version. All authors contributed to the article and approved the submitted version.

## Conflict of Interest

The authors declare that the research was conducted in the absence of any commercial or financial relationships that could be construed as a potential conflict of interest.

## Publisher's Note

All claims expressed in this article are solely those of the authors and do not necessarily represent those of their affiliated organizations, or those of the publisher, the editors and the reviewers. Any product that may be evaluated in this article, or claim that may be made by its manufacturer, is not guaranteed or endorsed by the publisher.

## Abbreviations

AD: Alzheimer's disease
AFT: Animal Fluency Task
ALS: Amyotrophic lateral sclerosis
BDT: Block Design test
BIS: Barratt Impulsivity Scale
BSIT: Brief-Smell Identification Test
COWAT: Controlled Oral Word Association Test
CWIT: Color word interference test
DAT: Delayed Alternation Task
DFC: Dynamic Functional Connectivity
DKEFS: Delis–Kaplan Executive Function System
DLPFC: Dorsolateral prefrontal cortex
DST: Digit symbol substitution tasks
DSST: Digit Symbol Substitution Test
FAB: Frontal Assessment Battery
FTD: Frontotemporal Dementia
IGT: Iowa Gambling Test
IST: Information Sampling Task
OCD: obsessive-compulsive disorder
OD: Olfactory dysfunction
OFC: Orbitofrontal cortex
PD: Parkinson's disease
PFC: Prefrontal cortex
PTSD: Post-traumatic stress disorder
ROCFT: Rey-Osterrieth Complex Figure Test
SCWT: Stroop Color and Word Test
SS: Sniffin Stick
SST: Stop-Signal task
TBI: Traumatic brain injuries
TMT: Trail Making Test
TMT-B: Trail Making Test-B
UPSIT: University of Pennsylvania Smell Identification Test
VD: vascular dementia
WAIS: Wechsler Adult Intelligence Scale
WCST: Wisconsin Card Sort Test.
