# Supplementary material for: Olfaction and Executive Cognitive Performance: A Systematic Review
Source: Front Psychol. 2022 May 9;13:871391. doi: 10.3389/fpsyg.2022.871391 (PMC9125097; doi:10.3389/fpsyg.2022.871391)
Supplement: Supplementary file 1 [file Table_1.docx]

**Table 1** Summary of the characteristics and main outcomes of the studies included in the systematic review.

| **Study** | **Study design** | **Sample description** | | | | | **Olfactory assessment** | **Neuropsychological assessment** | | **Olfactory-** **neuropsychological correlation** | |
| --- | --- | --- | --- | --- | --- | --- | --- | --- | --- | --- | --- |
|  |  | **N group** | **Age (years)** | **Male %** | **Education (years)** | **Country** |  | **Test/questionnaire** | **Executive function domain** | **Statistical results** | **Statistical test** |
| **TRAUMATIC INJURIES** | | | | | | | | | | | |
| Callahan and Hinkebein 1999 | Prospective quasi-experimental between-groups design | 68 TBI( Mild, mod, severe) | 32.61 + 13.02 | 80 | 12.25 + 1.69 | USA | University of Pennsylvania Smell Identification Test (UPSIT) | WAIS-R Digit symbol | Working memory | *P=*NS | Student’s *t*  test, Pearson correlation |
|  |  | 44 Mild Anosmic |  |  |  |  |  | TMT-B | Mental flexibility | *P*= .01 |  |
|  |  | 24 Normosmic | 30.79 + 11.70 | 67 | 11.46 + 1.91 |  |  | WCST | Mental flexibility | *P*= 0.003 |  |
|  |  |  |  |  |  |  |  | COWAT | Verbal fluency | *P*= NS |  |
| Sigurdardottir et al., 2010 | Prospective longitudinal | 40 Mild TBI | 35.9 ± 11.4 | 62.5 | 14.3 ± 2.5 | Norway | Brief Smell Identification Test | CWIT | Inhibition | *P = NS* | Spearman correlation (covariate: age) |
|  |  |  |  |  |  |  |  |  | Mental flexibility | *P = NS* |  |
|  |  | 34 Moderate TBI | 33.5 ± 10.8 | 73.52 | 12.5 ± 3.0 |  |  | D–KEFS (verbal fluency, category fluency, design fluency, semantic fluency) | Verbal fluency | *R = 0.22, p < 0.05* |  |
|  |  |  |  |  |  |  |  |  | Planning, | *P = NA* |  |
|  |  |  |  |  |  |  |  |  | mental flexibility |  |  |
|  |  |  |  |  |  |  |  |  | Nonverbal fluency | *P = 0.22, p < 0.05* |  |
|  |  | 41 Severe TBI | 28.5 ± 10.4 | 75.6 | 12.6 ± 1.9 |  |  | Iowa Gambling Task net score | Decision-making performance | *P = NS* |  |
| de Guise et al., 2015 | Cross-sectional | 68 TBI | 52.8 ± 21.1 | 62.9 | NA | Canada | Sniffin’ Sticks Test  (identification) | Battery of Cognitive | Verbal fluency | *r* = 0.30, *p* = 0.026 | Pearson’s correlation  (*α* = 0.05) |
|  |  |  |  |  |  |  |  | Efficiency Tests  WAIS - digit forward/backward recall | Working memory | Backward: *r* = 0.29, *p* = 0.033 |  |
|  |  |  |  |  |  |  |  | TMT B | Mental flexibility | *r* = -0.45, *p* = 0.004 |  |
| Osborne-Crowley and Skye McDonald, 2016 | Cross-sectional comparative | 23 TBI | 45.43 + 15.44) | 78.2 | 13.61 + 2.74 | Australia | The Brief Smell Identification Test | Observational measure of social disinhibition | Disinhibition | NS | Pearson correlations |
|  |  | 15 Control | 42.67 + 15.27 | 80 | 14.87 + 1..69 |  |  | Neuropsychiatric Inventory Disinhibition Domain |  |  |  |
| Sigurdardottir et al.,2016 | prospective population-based multicentre study | 129 TBI | 38.3 + 17.4 | 77 | 36% > 12 years | Norway | UPSIT  B-SIT | TMT Number–Letter Switching | Mental flexibility | In the B-SIT  TMT Number-Letter Switching task, *F*(2,  61) =7.79, *p* = .001,  the VFT Category Switching  task, *F*(2, 61) =4.37, *p* =.017, and the CVLT-II  long-delay free recall trial, *F*(2, 61)=3.19, *p* =.048,  nonsignificant correlation with UPSIT score (all *p* = 0.24, *n* = 65) and significantly correlated with the B-SIT score (*r* = .35 – .52, *p*s = .05, *n* = 64) | Multivariate analysis of variance  Kolmogorov–Smirnov test  Bonferroni-corrected level of significance  set at *p* <0.008 |
|  |  |  |  |  |  |  |  | VFT Letter Fluency, Category Fluency, Category Switching | Attentional Switching and fluency |  |  |
|  |  |  |  |  |  |  |  | CWIT: inhibition,, inhibition/Switching, Color Naming and Word Reading | Attentional Switching  Inhibition |  |  |
|  |  |  |  |  |  |  |  | TMT Visual Scanning, Number Sequencing, Letter Sequencing, Motor Speed | Processing sped |  |  |
| Bakker et al.,2017 | prospective longitudinal study | 27 children | 12.08 + 1.95 | 29.6% | NA | Australia | UPSIT | TMT-B, Creature Counting, CWIT | Attentional Switching | All: NORM and OD groups VF letter (Z = −1.701, p = .093)  VF category: Z = −2.254, p = .023.  Mean scores of EF was significant with VF(t (26) = 3.933, .  p = .001),  PTA duration and UPSIT T1 on  WDW at T2 was F (2, 23) = 8.672, p = .002. At T2, EF function (GEC) at T2, F(2,23) = 1.986,  p = .160. At T3,  EF.  At T3, F (2, 20) = 1.179, p = .328.  The independent effect of UPSIT T1  β = .379, p = .064, r2 change = 14%. The independent effect of PTA duration, β = -  .029, p = .884, r2 change = 1%.  At, 8 or 18 months after injury, acute olfactory function did not significantly predict EF outcomes. | Hierarchical multiple regression |
|  |  |  |  |  |  |  |  | Walk Don’t Walk, Colour Word | Inhibition |  |  |
|  |  |  |  |  |  |  |  | The global executive composite (GEC), behavioral  regulation index (BRI) and metacognition  index (MCI) | Behavioural EF |  |  |
|  |  |  |  |  |  |  |  | Letter fluency total and category fluency | Fluency |  |  |
|  |  |  |  |  |  |  |  |  |  |  |  |
| Giguère et al., 2019 | Cross-sectional | 20 Mild TBI | 33.05 ±12.24 | 60 | 13.45 ± 1.70 | Canada | Sniffin’ Sticks Test  (detection, discrimination, identification) | TMT B | Mental flexibility | *P = NS* | Spearman correlation |
|  |  |  |  |  |  |  |  | WAIS-IV digit span (backward) | Working memory | *P = NS* |  |
|  |  | 22 Orthopedic injury | 38.68 ± 13.31 | 41 | 14.50 ± 1.40 |  |  | Digit Span task and Visuospatial Index, attention index |  | *P = NS* |  |
| Langdon et al., 2021 | Prospective quasi-experimental between-groups design | 43 TBI OI | 34.7 ± 11.4 | 67 | NA | Spain | BAST-24 | TMT B | Mental flexibility | *P =* NS | Student’s *t* test |
|  |  |  |  |  |  |  |  | SCWT | Inhibitory control |  |  |
|  |  |  |  |  |  |  |  | WCST - category, perseverence | Working memory  processing |  |  |
|  |  | 68 TBI  no OI | 31.6 ± 10.2 | 72 |  |  |  | COWAT | Verbal fluency |  |  |
|  |  |  |  |  |  |  |  | WAIS – Digit Symbol, Block Design, Inverse Digit, Letter-Number Sequencing  Block design | Working memory  Visuospatial |  |  |
| **NEURODEGENERATIVE DISEASES** | | | | | | | | | | | |
| Westervelt et al., 2005 | Cross-sectional comparative | 74 memory concerns (63 probable dementia) | 70.3 ± 9.2 | 46.0 | 13.8 ± 3.2 | USA | Brief Smell Identification Test  (IB-SIT) (identification) | WMS - digit span | Working memory | All: *r* = 0.29, *p* < 0.010 | Pearson’s correlation/linear regression (covariates age and education)  (*α* = 0.01) |
|  |  |  |  |  |  |  |  | TMT B | Executive function | All: *r* = 0.38, *p* < 0.010; Patients: *r* = 0.32; Control: *r* = -0.01 |  |
|  |  |  |  |  |  |  |  | COWAT |  |  |  |
|  |  |  |  |  |  |  |  | ROCFT BQSS copy organization |  |  |  |
|  |  | 26 control |  |  |  |  |  | Animal Fluency | Verbal fluency | All: *r* = 0.54, *p* < 0.010; Patients: *r* = 0.44; Control: *r* = 0.71;  All: βs = 0.35, *p* < 0.009 |  |
| Lehner et al., 2009 | Cross-sectional | 11 MCI amnesic single domain | 69.6 ± 7.5 | 36.36 | 12 | Austria | UPSIT | WAIS-R digit symbol | Working memory | R = .30, *p* <0.01 | Partial Spearman correlation (covariates: age and MMSE) |
|  |  | 19 MCI amnesic multiple domain | 68.4 ± 9.0 | 42.1 | 10 |  |  | SCWT | Inhibitory control | R = -0.47, *p* < 0.001 |  |
|  |  | 21 MCI non-amnesic single domain | 64.6 ± 9.3 | 23.8 | 10 |  |  | Cerebral insufficiency test symbol counting | Working memory | R = -0.23, *p* < 0.05 |  |
|  |  | 13 MCI non-amnesic multiple domain | 64.1 ± 10.2 | 30.76 | 10 |  |  | cerebral insufficiency test interference | Inhibitory control | R = -0.28, *p* < 0.01 |  |
|  |  |  |  |  |  |  |  | TMT B | Mental flexibility | R = -0.26, *p* < 0.01 |  |
|  |  | 40 control | 66.5 + 8.2 | 47.5 | 10 |  |  | Maze test | Planning | P = NS |  |
| Pardini et al., 2009 | Cross-sectional comparative | 25 CBS | 62.0 ± 9.0 | 48.0 | 14.4 ± 3.0 | USA | UPSIT (identification) | DKEFS - sorting test | Executive function | CBS: *βs* = 0.05, *p* = 0.790; fvFTD: *βs* = -0.28, *p* = 0.430 | Linear regression  (*α* = 0.05) |
|  |  | 22 fvFTD | 60.3 ± 8.3 | 54.5 | 15.9 ± 3.0 |  |  |  |  |  |  |
|  |  | 14 control | NA | NA | NA |  |  |  |  |  |  |
| Bahar-Fuchs et al., 2010 | Cohort prospective | 14 AD | 73.0 ± 5.3 | 30.0 | 13.0 ± 2.3 | Australia | SIT-Smell identification test (identification errors) | DKEFS - category fluency | Verbal fluency | Right nostril: *r* = −0.34, *p* < 0.050 | Pearson’s correlation (*α* = 0.05) |
|  |  | 13 aMCI (6 with AD after 12 months) | 74.5 ± 7.1 | 53.8 | 14.8 ± 5.9 |  |  |  |  |  |  |
|  |  | 10 control | 74.1 ± 10.2 | 50.0 | 13.8 ± 4.5 |  |  |  |  |  |  |
|  |  |  |  |  |  |  |  | WAIS - digit span and digit symbol coding | Executive function | Right nostril: *r* = −0.38, *p* < 0.050 |  |
|  |  |  |  |  |  |  |  | ROCFT - copy |  |  |  |
|  |  |  |  |  |  |  |  | DKEFS - letter fluency |  |  |  |
| Morley et al., 2011 | Cross-sectional | 248 PD | 64+10 | 75% | 16 | USA | UPSIT (identification) | WAIS - forward digit span | Working memory | Between UPSIT (Upper and bottom) and Digit Span p = 0.008.  Digit Span (OR 1.0 (0.52–2.1) *p*=0.90), Significant Covariates  at Final Iteration: Hoehn/Yahr, age | Nonparametric Mann-Whitney tests and  Logistic regression  models |
|  |  |  |  |  |  |  |  | SCWT | Inhibitory control | Between UPSIT (Upper and bottom) and SCWT*, p* =0.001. SCWT( OR 1.3 (0.50–3.2). *p* =0.61, Significant Covariates  at Final Iteration Hoehn/Yahr, sex, PD duration |  |
|  |  |  |  |  |  |  |  | Tower of London-Drexel Test | Planning | Between UPSIT (Upper and bottom) and tower of London, *p* <0.001  Tower of London  ( OR 3.1 (1.5–6.2) *P*=0.001. Significant Covariates  at Final Iteration: UPSIT, Hoehn/Yahr. |  |
| Tkalčić et al., 2011 | Cross-sectional comparative | 15 probable AD | 77.8  ±  7.2 | 27.0 | NA | Croatia | Scandinavian Odor-Identification Test (identification) | Category Fluency Test | Verbal fluency | AD: category/semantic fluency *r* = 0.74, *p* < 0.01; VD: NS; Control: NS | Pearson’s correlation (*α* = 0.05) |
|  |  | 11 VD |  | 27.0 |  |  |  |  |  |  |  |
|  |  | 30 control | 79.8 ± 5.9 | 33.0 |  |  |  |  |  |  |  |
| Parrao et al., 2012 | Cross-sectional comparative | 44 PD | 63.6  ±  10.2 | NA | 11.3 ± 3.5 | Chile | Sniffin’ Sticks Test (identification) | WMS - digit span forward/backward | Working memory | NS | Spearman’s correlation (*α* = 0.05) |
|  |  |  |  |  |  |  |  | WCST | Mental flexibility | Correct answers *r* = 0.23, *p* < 0.005 |  |
|  |  |  |  |  |  |  |  | TMT B | Mental flexibility | NS |  |
|  |  |  |  |  |  |  |  | ROCFT-copy | Visuospatial processing | NS |  |
|  |  | 17 control | 63.2  ±  9.5 | NA | 13.2 ± 2.8 |  |  | Tower of London | Planning | NS |  |
|  |  |  |  |  |  |  |  | Semantic/phonological verbal fluency | Verbal fluency | NS |  |
|  |  |  |  |  |  |  | Olfactory Detection Threshold (vanillin and propionic acid) | WMS - digit span forward/backward | Giguère et al., 2019mory | NS |  |
|  |  |  |  |  |  |  |  | WCST | Mental flexibility | NS |  |
|  |  |  |  |  |  |  |  | TMT B | Mental flexibility | NS |  |
|  |  |  |  |  |  |  |  | ROCFT-copy | Visuospatial processing | NS |  |
|  |  |  |  |  |  |  |  | Tower of London | Planning | NS |  |
|  |  |  |  |  |  |  |  | Semantic/phonological verbal fluency | Verbal fluency | NS |  |
| Hanoglu et al., 2014 | Cross-sectional comparative | 10 PD-stage 1 | 59.6  ±  7.0 | 90.0 | NA | Turkey | UPSIT (identification) | SCWT | Inhibitory control | PD: Time *r* = -0.31, *p* = 0.033; Error *r* = -0.30, *p* = 0.037 | Pearson/Spearman’s correlation (*α* = 0.05) |
|  |  | 18 PD-stage 2 | 67.9  ±  6.0 | 77.8 | NA |  |  |  |  |  |  |
|  |  | 19 control | 62.8  ±  6.0 | 57.9 | NA |  |  |  |  |  |  |
|  |  |  |  |  |  |  |  | Categorical Verbal Fluency Test | Verbal fluency | PD: *r* = 0.18, *p* = 0.215 |  |
|  |  |  |  |  |  |  |  | Benton Face Recognition Test | Visuospatial processing | PD: *r* = 0.11, *p* = 0.462 |  |
|  |  |  |  |  |  |  |  | Benton Line Judgment Orientation Test | Visuospatial processing | PD: *r* = 0.10, *p* = 0.511 |  |
| Kjelvik et al., 2014 | Cohort prospective | 18 patients (6 AD and 12 aMCI (13 AD and 5 aMCI after 0.5-1.5 years)) | 74.6  ±  6.3 | 44.4 | 15.3 ± 2.5 | Norway | Brief Smell Identification Test (identification) | TMT-B | Mental flexibility | P = NS | Student’s t-test  (for scoring >50% or ≤50% on olfaction measure across patients) |
|  |  |  |  |  |  |  |  | ROCFT copy | Visuospatial processing | P = NS |  |
|  |  | 30 control | 67.4  ±  7.6 | 53.3 | 17.1 ± 3.5 |  | Sniffin’ Sticks Identification Test (identification and discrimination) | TMT-B | Mental flexibility | P = NS |  |
|  |  |  |  |  |  |  |  | ROCFT copy | Visuospatial processing | P < 0.05 |  |
| Devanand et al., 2015 | Cohort prospective | 130 aMCI  (42 with dementia at 2-4 years follow-up) | 81.2  ±  5.6 | 25.4 | 11.0 ± 4.1 | USA | UPSIT (identification) | Naming, letter, and category fluency | Verbal fluency | Letter fluency *r* = 0.28, *p* < 0.0001;  Category fluency *r* = 0.31, *p* < 0.0001; Naming *r* = 0.22, *p* < 0.0001 | Spearman’s correlation (*α* = 0.05) |
|  |  | 125 naMCI (32 with dementia at 2-4 years follow-up) | 82.2  ±  6.5 | 25.6 | 7.3  ±  4.8 |  |  |  |  |  |  |
|  |  | 498 control (35 with dementia at 2-4 years follow-up) | 79.3  ±  5.0 | 31.1 | 11.5  ±  4.7 |  |  |  |  |  |  |
| Vyhnalek et al., 2015 | Cross-sectional comparative | 75 aMCI | 72.7 ± 9.1 | 48.0 | 14.0 ± 3.4 | Czech Republic | Motol Hospital Smell Test (identification) | ROCFT copy | Visuospatial processing | All: NS; aMCI: *β* = 1.44, *p* = 0.002; naMCI: NS; Control: NS | Ordinary least squares regression (covariates age and gender; *α* = 0.05) |
|  |  | 32 naMCI | 69.2 ± 9.9 | 46.9 | 15.5 ± 2.7 |  |  |  |  |  |  |
|  |  | 26 mild probable AD | 74.2 ± 8.0 | 23.1 | 12.0 ± 3.0 |  |  | Digit forward and backward | Working memory | All: NS; aMCI:  *β* = 0.06, NS; naMCI: NS; Control: NS |  |
|  |  | 27 control | 69.1 ± 8.5 | 22.2 | 15.2 ± 2.7 |  |  | Category Fluency and  Initial Letter Fluency test | Executive function | All: NS; aMCI:  *β* = -0.01, NS; naMCI: NS; Control: NS |  |
|  |  |  |  |  |  |  |  | TMT B |  |  |  |
| Orasji et al., 2016 | Cross-sectional comparative | 9 bvFTD | 73.1 ± 10.3 | 88.9 | NA | The Netherlands | Brief Smell Identification Test  (identification) | Frontal Assessment Battery | Executive function | NS | Spearman’s correlation (*α* = 0.05) |
|  |  | 11 control | 71.6 ± 6.1 | 54.5 | NA |  | Odor and Semantics Battery (identification, discrimination, and association) | Frontal Assessment Battery | Executive function | NS |  |
| Pilotto et al., 2016 | Cross-sectional comparative | 28 ALS | 68.6±9.7 | 45.1 | 8.1 ± 4.5 | Italy | Sniffin’ Sticks Test (identification) | TMT B | Mental flexibility | NS | Pearson’s Partial Correlation (covariate age; *α* = 0.05) |
|  |  | 30 control | 67.5 ± 9.7 | NA | NA |  |  |  |  |  |  |
|  |  |  |  |  |  |  |  | Phonemic and  semantic fluency test | Verbal fluency | NS |  |
|  |  |  |  |  |  |  |  | ROCFT  Frontal Assessment Battery | Visuospatial processing  Executive function | NS |  |
| Ward et al., 2016 | Cross-sectional comparative | 13 AD | 76.77+ 6.44 | 30 | 16.08 + 3.59 | Australia | UPSIT (identification)  ------------------  The Odor Memory Test is a 12-item four-alternative  forced-choice test with microencapsulated  odorants in scratch and sniff format | Iowa Gambling Task net score | decision-making performance | IGT net score, *P*= .104.  IGT shifting decks, p=<.05;  BART,P=0.943  Pairwise comparison p and effect sizes AD vs. HE, p < .05, d = −1.21 | Bivariate Pearson correlations  Hierarchical multiple regression models |
|  |  | 8 MCI | 76.13 + 6.29 | 62.5 | 15.5 + 3.51 |  |  | Iowa Gambling Task shifting decks | decision-making performance |  |  |
|  |  | 20 control | 76.65 + 6.48 | 55 | 16.98 + 2.80 |  |  | The Balloon Analogue Risk Task(BART) | Impulsivity |  |  |
| Goette et al., 2017 | Cross sectional | 70 dementia patients | 69.7 ± 9.7 | 42.85% | 14.6 ± 2.8 | USA | UPSIT | TMT B, RBANS battery | Mental flexibility | UPSIT raw: r = 0.37, p < 0.01 ; UPSIT t: r = 0.34, p < 0.01 | Pearson correlation |
|  |  |  |  |  |  |  |  | SCWT (Word, Color, Interference) | Inhibitory control | All p = NS (Word, Color, Interference & UPSIT raw, UPSIT t) |  |
|  |  |  |  |  |  |  |  | WCST ( Perseverative Responses, Categories Completed, Failure to Maintain Set) | Mental flexibility | UPSIT raw & Perseverative Responses: r = 0.21, p < 0.05 ; UPSIT t & Perseverative Responses: r = 0.29, p < 0.01;  UPSIT raw &: Categories Completed: r = 0.24, p < 0.05; UPSIT t & Categories Completed: r = 0.24, p < 0.05;  UPSIT raw & Failure to Maintain Set: r = -0.26, p < 0.05; UPSIT t & Failure to Maintain Set: r = -0.26, p < 0.05 |  |
|  |  |  |  |  |  |  |  | Iowa Gambling Task, RBANS battery, total net | decision-making performance | UPSIT raw: r = 0.24, p < 0.05 ; UPSIT t: r = 0.15, p = NS |  |
|  |  |  |  |  |  |  |  | Phonemic Fluency (FAS, RBANS battery) | Verbal fluency | UPSIT raw score: r = 0.22, p < 0.05; UPSIT t score: r = 0.37, p < 0.01 |  |
|  |  |  |  |  |  |  |  | RBANS (Category Fluency (Animals, RBANS battery) | Verbal fluency | UPSIT raw score: r = 0.2, p = NS; UPSIT t score: r = 0.31, p < 0.01 |  |
|  |  |  |  |  |  |  |  | RBANS- Visuospatial/Constructional | Visualspatial processing | UPSIT raw score: r = .26, p < 0.05; UPSIT t score: r = 0.3, p < 0.01 |  |
| Carotenuto et al., 2018 | Cross-sectional comparative | 55 MS | 45.9 ± 14.2 | 31.0 | NA | Italy | UPSIT (identification | Symbol Digit  Modalities Test  COWAT | Processing speed  Verbal fluency | *r* = 0.58, *p* < 0.001  Phonemic *r* = 0.57, *p* < 0.001 | Partial correlation (covariates age, gender, education,smoking, and smell dysfunction awareness; *α* = 0.004) |
|  |  | 20 control | 40.1 ± 13.0 | 50.0 |  |  |  |  |  |  |  |
| Chen et al., 2018 | Cross-sectional comparative | 125 LLD | 66.7 ± 6.2 | 23.2 | 8.0 ± 4.0 | China | Sniffin’ Sticks Test (identification) | Stroop A | Inhibitory control | Across LLD: r = -0.223, p = 0.028; LDD with OII > LLD without OII, p < 0.05;  AD with OII vs AD without OII, p = NS; controls OII vs controls without OII, p = NS | Pearson correlation; ANOVA |
|  |  | 50 AD | 71.9 ± 9.9 | 44 | 6.8 ± 4.1 |  |  | Stroop Interference Effect |  | Across LLD: p = NS;  LDD with OII vs LLD without OII, p = NS;  AD with OII vs AD without OII, p = NS; controls with OII vs controls without OII, p = NS |  |
|  |  | 60 controls | 65.4 ± 7.3 | 40 | 9.1 ± 3.5 |  |  | TMT B | Mental flexibility | Across LLD: p = NS;  LDD with OII vs LLD without OII, p = NS;  AD with OII vs AD without OII, p = NS; controls with OII vs controls without OII, p = NS; controls without OII < LLD with OII, p < 0.05 |  |
|  |  |  |  |  |  |  |  | Clock Drawing Test and ROCF | Visual-space | Across LLD and Across groups: P=NS |  |
| Park et al., 2018 | Cross-sectional comparative | 17 SMI | 69.4 ± 8.3 | 5.9 | 9.3 ± 5.1 | Republic of Korea | CCSIT (identification) | Digit span forward and backward | Working memory | Forward *r* = 0.02, *p* = 0.809;  Backward *r* = 0.18, *p* = 0.052 | Spearman’s correlation (*α* = 0.05) |
|  |  | 50 aMIC | 73.0 ± 7.6 | 42.0 |  |  |  |  |  |  |  |
|  |  |  |  |  |  |  |  | ROCFT copy | Visuospatial processing | *r* = 0.23, *p* = 0.013 |  |
|  |  | 28 naMCI | 70.6 ± 7.3 | 10.7 |  |  |  |  |  |  |  |
|  |  | 20 AD | 75.5 ± 7.7 | 35.0 |  |  |  |  |  |  |  |
|  |  |  |  |  |  |  |  | COWAT | Verbal fluency | Semantic *r* = 0.51, *p* < 0.001; Phonemic *r* = 0.246, *p* = 0.011 |  |
|  |  |  |  |  |  |  |  | SCWT | Inhibitory control | Word *r* = 0.32, *p* = 0.001; color *r* = 0.31, *p* = 0.001 |  |
| Blanco et al., 2019 | Cross-sectional comparative | 146 Fibromyalgia | 51.21 + 9.22 | 0% | NA | Sprain | Connecticut Chemosensory Clinical Research Center Test | WAIS | working memory  . | Processing speed (p = .004) in comparing with control. Working memory was not significant. | Student’s t tests |
|  |  | 122 control |  |  |  |  |  |  | Processing speed |  |  |
| Lian et al., 2019 | Cross-sectional comparative | 30 AD-OD | 66.43 ± 11.71 | 33.3 | NA | China | Sniffin’ Sticks Test (composite) | SCWT | Inhibitory control | P = NS | Student’s *t* test  (*α* = 0.05) |
|  |  | 30 AD- without OD | 65.33 ± 9.99 | 43.3 | NA |  |  | ROCF | Visuospatial ability | P=0.007 |  |
| Mertens et al., 2019 | Cross-sectional | 198 Healthy controls | 67.4 ± 11.0 | NA | NA | Worldwide | UPSIT (identification | Montreal Cognitive Assessment Test (MoCA) subscore for visuospatial and executive function | Executive function | R = 0.13, p < 0.001 | Pearson correlation |
|  |  | 310 Asymptomatic genetic PD | 63.9 ± 7.4 |  |  |  |  |  |  |  |  |
|  |  | 220 Symptomatic genetic PD | 64.4 ± 10.2 |  |  |  |  |  |  |  |  |
|  |  | 491 Sporadic PD | 67.8 ± 9.8 |  |  |  |  |  |  |  |  |
|  |  | 61 Possible prodromal PD | 72.8 ± 6.1 |  |  |  |  |  |  |  |  |
| Velayudhan et al., 2019 | Cross-sectional comparative | 19 eoAD | 59.39 ± 5.3 | 63.2 | 11.59 ± 3.4 | UK | UPSIT (identification | CAMCOG (subscale) | Executive function | *r* = 0.508, *p* = 0.026 | Pearson correlation (*α* = 0.05) |
|  |  | 17 eoMCI | 58.35 ± 4.1 | 64.7 | 12.56 ± 2.7 |  |  |  |  |  |  |
|  |  | 21 control | 60.7 ± 11.2 | 52.4 | 15.5 ± 3 |  |  |  |  | NA | NA |
| Yoshii et al., 2019 | Cross sectional | 48 PD | 66 ± 10 | 41.66% | NA | Japan | Odor Stick Identification Test for Japanese (OSIT-J) | WCST (sub-scores for categories achieved (CA), perseverative errors of Nelson type (PEN), and difficulties of maintaining set (DMS)) | Mental flexibility | WCST categories achieved : r = 0.317, p = 0.028; WCST perseverative errors of Nelson type: r = − 0.203, p=NS; WCST difficulties  of maintaining set:r=−0.322,p=0.026 | Spearman correlation |
| Yoo et al., 2019 | Retrospective cohort study | 15 Normosmic PD | NA | 40 | 9 | South Korea | CCSIT | COWAT and clock drawing test | frontal executive domain | All p = NS  Longitudinal changes of cognition in patients with PD over 5 years was significant for executive domain between groups of PD. p=0.008 | Kruskal–Wallis test and Kaplan –Meier analysis |
|  |  | 40 hyposmic patients with  PD without olfactory anosognosia |  | 42.5 | 9 |  |  | ROCFT copy and pentagon  drawing test | visuospatial domain |  |  |
|  |  | 22 hyposmic patients with PD with olfactory anosognosia |  | 50 | 8 |  |  | digit span task and SCWT | attention  domain |  |  |
| Tahmasebi et al., 2020 | Cross-sectional comparative | 163 Patients with aMCI | 69 | 46.6 | 12 | Austria | Sniffin’ Sticks | TMT B-A | Mental flexibility | P= < 0.001 between overrater, correct normosmic, Correct hyposmic and underrater groups. | Kruskal-Wallis and Pearson’s chi-squared  test. |
|  |  | 210 Patients with naMCI | 69 | 64.3 | 11 |  |  |  |  |  |  |
|  |  | 38 Patients with AD | 74.5 | 44.7 | 9 |  |  |  |  |  |  |
|  |  | 69 Patients with SCD | 66 | 49.3 | 12 |  |  |  |  |  |  |
|  |  | 161 Control subjects | 74.5 | 37.3 | 11 |  |  |  |  |  |  |
| Yoo et al., 2020 | Retrospective cohort study | 44 Normosmic PD | 62.7 ± 8.4 | 43.2 | NA | South Korea | CCSIT | Digit span forward and backward | Working memory | All p = NS | ANCOVA (covariates: age, sex, and the interval from PD onset) |
|  |  | 136 Hyposmic PD | 64.9 ± 7.8 | 39 |  |  |  | COWAT semantic and phonemic | Verbal fluency |  |  |
|  |  | 48 Anosmic PD | 69.7 ± 6.8 | 66.7 |  |  |  | ROCFT copy | Visuospatial domain |  |  |
| Wang et al., 2021 | Cross-sectional | Controls | 67.5 ± 5.3 | 51.4% | 11.8 ± 2.9 | China | Sniffin’ Sticks Test (detection, discrimination, and identification | TMT B | Executive function | All p = NS (across all subjects and within subjective cognitive decline and MCI groups) | Partial correlation (covariates: age, sex, and years of education) |
|  |  | 84 SCD | 67.0 ± 5.6 | 38.1% | 11.5 ± 3.3 |  |  |  |  |  |  |
|  |  | 129 MCI | 67.8 ± 8.6 | 31.8% | 9.8 ± 3.2 |  |  | ROCF | Visuospatial skill |  |  |
|  |  | 52 AD | 71.2 ± 10.3 | 44.2% | 8.0 ± 4.1 |  |  |  |  |  |  |
| **PSYCHIATRIC DISORDER** | | | | | | | | | | | |
| Seidman et al., 1992 | Cross-sectional comparative study | 16 schizophrenia  17 control | 36.5 + 8. I | 93.75 | 12.1 + 2.5 | USA | UPSIT (identification  ) | WCST ( category and perseverations) | Mental flexibility | Schizophrenics were significantly more  Impaired than controls on all three measures: UPSIT (I= -3.94, df=31, p<0.001): WCS7 categories (J= -4.97. df=30. P=<0.001):  WCST perseverations: / = 3. IS. df= 30. /, p=< 0.005). | Pearson’s product correlation |
|  |  |  | 31.7 + 8.9 | 93.75 | 15.7 + 2.2 |  |  |  |  |  |  |
| Brewer et al., 1996 | Cross-sectional comparative | 27 schizophrenia | 31.8 ± 8.5 | 100.0 | NA | Australia | UPSIT (identification | Modified WCST | Mental flexibility | Schizophrenia: categories *r* = 0.56, *p* < 0.050; perseverations *r* = -0.33, NS; set loss *r* = -0.31,  NS; Control: categories *r* = 0.40, NS; perseverations *r* = -0.23, NS; set loss *r* = -0.31, NS  Schizophrenia: error *r* = -0.01, NS; perseverations *r* = 0.11, NS; Control: error *r* = -0.26, NS; perseverations *r* = -0.21, NS  Schizophrenia: trials-to-criterion *r* = -0.33, NS; error *r* = -0.39, NS; Control: trials-to-criterion *r* = -0.00, NS; error *r* = 0.00, NS | Pearson’s correlation (*α* = 0.05) |
|  |  |  |  |  |  |  |  | Delayed alternation task | Working memory |  |  |
|  |  | 19 control | 34.8 ±  12.5 | 100.0 |  |  |  | Delayed response task | Working memory |  |  |
|  |  |  |  |  |  |  |  |  |  |  |  |
| Seidman et al., 1997 | Cross-sectional comparative | 40 schizophrenia | 38.5 ± 6.5 | 60.0 | 13.2 ± 1.9 | USA | UPSIT (identification) | WCST | Mental flexibility | Schizophrenia: categories *r* = 0.30, NS; perseverations *r* = 0.04, NS | Pearson’s correlation  (*α* = 0.05) |
|  |  | 32 control | 36.4 ± 9.3 | 46.9 | 14.8 ± 2.6 |  |  | WAIS - Block Design | Visuospatial processing | Schizophrenia: NS |  |
| Purdon, 1998 | Cross-sectional | 21 schizophrenia | 37.1 ± 9.6 | 85.7 | 12.1 ± 2.3 | Canada | UPSIT (identification | SCWT | Inhibitory control | colour naming speed *r* = 0.57, *p* = 0.006; incongruous colour naming speed *r* = 0.63, *p* = 0.002; interference index *r* = 0.44, *p* = 0.047 | NA |
| Saoud et al., 1998 | Cross-sectional comparative | 24 schizophrenia | 31.3 ± 8.9 | 100.0 | 11.7 ± 2.6 | France | Sentosphère olfactory test (identification) | WCST | Mental flexibility | Schizophrenia: forced choice identification - categories *r* = 0.24, NS; perseverative errors *r* = -0.24, NS; spontaneous identification -categories *r* = 0.31, NS; perseverative errors *r* = -0.54, *p* < 0.050; control: forced choice identification NA; spontaneous identification -categories *r* = -0.17, NS; perseverative errors *r* = -0.35, NS | Spearman’s correlation |
|  |  | 21 control | 30.2 ± 9.2 | 100.0 | 14.5 ± 3.9 |  |  |  |  |  |  |
| Barnett et al., 1999 | Cross-sectional comparative | 20 OCD | 37.34 + 12.2 | 40 | 6.25 + 1.9 | Australia | UPSIT | Spatial working memory  Spatial recognition | Working memory  Visuospatial processing | OCD: NS; Control: NS  Between OCD and Control: p= <0.05 | Pearson's product  moment correlation. |
|  |  | 23 control | 37.65 + 14.5 | 43 | 5.52 + 1.9 |  |  |  |  |  |  |
| Vasterling et al., 2000 | Prospective study | 26 Deployed PTSD | 50 + 3.7 | 100% | 12.8 +2.5 | USA | UPSIT (identification) | WCST | Mental flexibility | P = NS | Pearson correlation |
|  |  | 25 Non-deplyed non-PTSD | 51.4 +5.3 |  | 14.7 + 2.3 |  |  |  |  |  |  |
|  |  | 17 Non-deployed | 47 + 4.7 |  | 14.1 + 1.9 |  |  |  |  |  |  |
| Corcoran et al., 2005 | Cross-sectional | 26 early psychotic disorders | 15.0 ± 1.8 | 61.5 | NA | USA | UPSIT  (identification) | Phonemic Verbal Fluency | Verbal fluency | *r* = 0.67, *p* = 0.002 | Pearson’s correlation (*α* = 0.05) |
|  |  |  |  |  |  |  |  | Animal Naming | Verbal fluency | *r* = 0.62, *p* = 0.004 |  |
|  |  |  |  |  |  |  |  | Block design | Visuospatial processing | *r* = 0.08, *p* = 0.700 |  |
|  |  |  |  |  |  |  |  | SCWT | Inhibitory control | *r* = 0.55, *p* = 0.020 |  |
|  |  |  |  |  |  |  |  | TMT B | Mental flexibility | *r* = -0.51, *p* = 0.020 |  |
| Rupp et al., 2006 | Cross-sectional comparative | 32 alcohol-dependent | 44.6 ± 10.7 | 56.0 | 9.5 ± 1.6 | Austria | Sniffin’ Sticks Test (detection, discrimination, and identification) | WCST | Mental flexibility | Discrimination: alcohol-dependent *r* = 0.52, *p* = 0.002; control: NS; all: *β* = 0.39; *p* = 0.001; Detection/identification: NS | Pearson’s correlation/linear regression  (*α* = 0.05) |
|  |  | 30 control | 45.3 ± 8.7 | 53.0 | 10.0 ± 2.5 |  |  |  |  |  |  |
| Dileo et al., 2008 | Cross-sectional comparative | 31 PTSD | 58.2 ± 2.6 | 100.0 | NA | Australia | UPSIT  (identification) | Eysenck's Impulsivity Questionnaire | Impulsivity | *r* = -0.31, *p* < 0.05 | Pearson’s correlation (*α* = 0.05) |
|  |  | 31 control | 56.8 ± 7.2 | 100.0 | NA |  |  |  |  |  |  |
| Maurage et al., 2011 | Cross-sectional comparative | 20 alcohol-dependent | 50.2 ± 11.8 | 55.0 | 15.1 ± 3.4 | Belgium | Sniffin’ Sticks Test (detection, discrimination, and identification) | Stop-Signal task | Inhibitory control | Threshold: control *r* = -0.35, NS; alcohol-dependent *r* = -0.15, NS; Discrimination: control *r* = -0.02, NS; alcohol-dependent *r* = -0.33, NS; Identification: control *r* = 0.14, NS; alcohol-dependent *r* = -0.02, NS | Pearson’s correlation (*α* = 0.05) |
|  |  |  |  |  |  |  |  | Stop-Signal task | Inhibitory control | Control *r* = 0.11, NS; alcohol-dependent *r* = -0.22, NS; |  |
|  |  | 20 control | 47.5 ± 9.7 | NA | 15.4 ± 3.2 |  | Retronasal test (identification) |  |  |  |  |
| Segalas et al., 2011 | Cross-sectional comparative study | 29 OCD  17 control | 35.57 + 11.5  31.7 + 8.9 | 51.72%  93.75 | 12.2 + 73.0  15.7 + 2.2 | Sprain | Sniffin’ Sticks  (detection, discrimination, and identification) | WAIS - Digit Span | Attention: | For OCD,  Olfactory identification  ( *β* -= - 1.13 p=. 0.01) and Olfactory discrimination  (*β* -=2.03 p=.< 0.0001)  For control, Olfactory identification and digit span, r= -0.5, p= <0.01. | Pearson’s correlations |
| Hardy et al., 2012 | Cross-sectional comparative | 20 bipolar disorder | 34.5 ± 8.9 | 25.0 | NA | USA | Smell Threshold Test (detection) | TMT B | Mental flexibility | Bipolar: NS | Pearson’s correlation (Fishers’s z score; *α* = 0.01) |
|  |  | 44 control | 32.6 ± 11.8 | 40.9 | NA |  | UPSIT  (identification) | TMT B | Mental flexibility | Bipolar: NS |  |
| Bersani et al., 2013 | Cross-sectional comparative | 25 OCD | 36.4 ± 12.3 | 24.0 | 13.8 ± 3.2 | Italy | Brief Smell Identification Test (identification) | Intra/  Extra Dimensional Set Shifting | Mental flexibility | OCD: NS; control: NS | Partial correlation(covariates age, gender, smoking status, and intelligence [also symptom severity for OCD]; *α* = 0.01) |
|  |  | 21 control | 36.8 ±13.3 | 47.6 | 14.9 ± 4.1 |  |  |  |  |  |  |
|  |  |  |  |  |  |  |  | Stop Signal Task | Inhibitory control | OCD: *r* = −0.615, *p* = 0.004; Control: NS |  |
|  |  |  |  |  |  |  |  | Information Sampling Task | Impulsivity | OCD: NS; Control: NS |  |
| Takahashi et al., 2018 | Cross-sectional comparative | 59 schizophrenia | 29.2 ± 8.6 | 50.8 | NA | Japan | T&T olfactometer | COWAT | Verbal Fluency | *rho =* − 0.245, *p=*  0.061 | Kolmogorov–Smirnov test) and Bonferroni’s correction for multiple comparisons |
|  |  | 32 at risk mental state | 17.6 ± 3.0 | 56.25 |  |  |  |  |  |  |  |
|  |  |  |  |  |  |  |  | Tower of London | Executive function | *rho =*0.150, *p=*0.255 |  |
|  |  | 169 control | 26.6 ± 5.6 | 57 |  |  |  |  |  |  |  |
| **CONTROL SAMPLES** | | | | | | | | | | | |
| Spinella, 2002 | Cross-sectional | 31 control | 30.6 ± 16.4 | 35.5 | 14.2 ± 1.6 | USA | Alberta Smell Test (identification) | Go/No-go task | Inhibitory control | Left nostril: conflict *r* = -0.49, *p* < 0.010 | Pearson’s correlation (*α* = 0.05) |
|  |  |  |  |  |  |  |  | Delayed alternation task | Working memory | Right nostril: trials-to-criterion *r* = 0.41, *p* < 0.05 |  |
| Vasterling et al., 2003 | Prospective study | 72 Deployed | 39.58 + 8.22 | 87.5 | 14.58 ± 2.03 | USA | UPSIT | WAIS-R Digit Span: forward and backward) | Working memory | All P = NS  All P = NS | standardized partial correlation (covariates: age, rank, Mississippi Scale, BDI, war-zone |
|  |  | 33 Non-deployed | 37.97 + 11.42 | 81.8 | 14.73 ± 1.92 |  |  | WCST: trials to completion, perseverations, failures to maintain set | Mental flexibility |  |  |
| Larsson et al., 2004 | Cross-sectional | 1906 control | NA | 45.5 | NA | Sweden | Scandinavian Odor-Identification Test (identification) | Letter–Digit Substitution test | Executive function | *r* = 0.34, *p* < 0.01; *β* = 0.13, *p* = 0.001 | Correlation/linear regression (covariates age, gender, and education) (*α* = 0.05) |
|  |  |  |  |  |  |  |  | Letter fluency | Verbal fluency | *r* = 0.25, *p* < 0.01 |  |
|  |  |  |  |  |  |  |  | Category fluency | Verbal fluency | *r* = 0.15, *p* < 0.01 |  |
|  |  |  |  |  |  |  |  | WAIS - Block Design | Visuospatial processing | *r* = 0.26, *p* < 0.01 |  |
|  |  |  |  |  |  |  |  | Tower of Hanoi | Planning | *r* = -0.11, *p* < 0.01 |  |
| Killgore and Mcbride, 2006 | Cross-sectional | 38 controls | 23.3 ± 3.7 | 57% | NA | USA | Smell Identification Test (SIT) | Color Trails Test (CTT) | Executive function | P = NS | Pearson correlation |
| Devanand et al., 2010 | Cross-sectional comparative | 170 aMCI | 82.0 ± 6.2 | 22.0 | NA | USA | UPSIT  (identification) | Category Fluency  Animal naming | Verbal fluency | Naming mean *r* = 0.31, *p* < 0.0001; Animal naming *r* = 0.28, *p* < 0.0001 | Spearman’s correlation (*α* = 0.05) |
|  |  | 120 naMCI | 80.7 ± 5.9 | 25.0 | NA |  |  |  |  |  |  |
|  |  | 802 control | 80.2 ± 5.7 | 31.0 | NA |  |  |  |  |  |  |
| Hedner et al., 2010 | Cross-sectional | 170 control | 57.2 ± 13.8 | 37.6 | NA | Germany | Sniffin’ Sticks Test (detection, discrimination, and identification) | Digit Span Backward Test | Working memory | Threshold: *r* = 0.13, NS; *βs* = -0.01, *p* = 0.877; Discrimination *r* = 0.08, NS; *βs* = -0.23, *p* = 0.006; Identification *r* = 0.02, NS; *βs* = -0.22, *p* = 0.013 | Correlation/linear regression (covariates age and gender)  (*α* = 0.05) |
|  |  |  |  |  |  |  |  | Letter fluency test | Verbal fluency | Threshold: *r* = 0.07, NS; *βs*=0.15, *p* = 0.097; Discrimination: *r* = 0.35, *p* < 0.01; *βs* = 0.37, *p* < 0.010; Identification *r* = 0.09, NS; *βs* = 0.18, *p* = 0.043 |  |
| Killgore et al., 2010 | Cohort prospective | 54 control | 23.5 ± 4.0 | 53.7 | NA | USA | Smell Identification Test (identification score change after sleep deprivation) | WCST | Mental flexibility | correct card placements *r* = 0.35, *p* = 0.01; completed sorting categories *r* = 0.31, *p* = 0.028; conceptual level responses *r* = 0.35, *p* = 0.014; non-perseverative errors *r* = -0.47, *p* = 0.001 | Partial correlation  (covariate stimulant medication; *α* = 0.05) |
| Lee et al., 2012 | Cross-sectional | 44 healthy right-handed female participants | 31.53 + 7.15 | 0% | 15.24 + 1.95 | China | Sniffin' Sticks | Risky Gains Task | Risk taking and impulsivity | For safe choice (p > 0.05,  d = 0.57). Total frequency of the risky choices of p > 0.05, d = 0.11. HOI scored significantly  higher than the LOI group on the scores of risk taking for SRT total= (t[42] = 2.21, p = 0.03, d = 0.67).  In the Choice RT  (t[42] = –0.62, p > 0.05, d = 0.19). In the Suppress  task,  (t[42] = 0.04,  p > 0.05, d = 0.01) | Independent-samples t-tests. A repeated-measures analysis of variance  (ANOVA) |
|  |  |  | 36.52 + 10.18 |  | 15.67 + 1.94 |  |  | Rotman-Baycrest  Battery to Investigate Attention | Attention |  |  |
| Bettison et al., 2013 | Cross-sectional | 59 control | NA | 49.1 | NA | Australia | Sniffin’ Sticks Test (discrimination and identification) | Iowa Gambling Task | Decision-making | Discrimination: total risk: *r* = -0.43, *p* < 0.01; risk avoidance *r* = -0.23, NS; Identification: total risk: *r* = -0.20, NS; risk avoidance *r* = 0.08, NS | Partial correlations (covariates gender, intelligence, and olfactory detection; *α* = 0.017-0.033) |
| Fagundo et al., 2015 | Cross-sectional comparative | 31 hyposmia control | 25.4 ± 8.6 | 0.0 | 15.6 ± 2.5 | Spain | Sniffin’ Sticks Test (discrimination and identification) | WCST | Mental flexibility | Identification *β* = -0.16-1.02, *p* = 0.313-0.872; Discrimination *β* =-1.28-0.65, *p* = 0.206-520 | Linear regression (covariates age and education; *α* = 0.05) |
|  |  | 31 normosmia control | 26.4 ± 8.4 | 0.0 | 16.8 ± 2.9 |  |  | SCWT | Inhibitory control | Interference: Identification *β* = 0.29, *p* = 0.034; Discrimination *β* = -0.05, *p* = 0.717 |  |
|  |  |  |  |  |  |  |  | Iowa Gambling Task | Decision-making | Interference: Identification *β* = 0.22, *p* = 0.066; Discrimination *β* = 0.04, *p* = 0.333 |  |
| Alosco et al., 2017 | Cross-sectional comparative | 95 former football players (self-reported cognitive, behavioral, and mood symptoms) | 55.3 ± 7.9 | 100.0 | 16.4 ± 1.0 | USA | Brief Smell Identification Test (identification) | TMT B | Executive function | Players: *r* = 0.22, *p* = 0.046 (TMT B *p* = 0.013; Digit Symbol Coding *p* = 0.017) | Partial  Correlations (covariates age and education; *α* = 0.05) |
|  |  |  |  |  |  |  |  | WAIS - Digit Span and Digit Symbol Coding |  |  |  |
|  |  | 28 control | 57.1 ± 6.9 | 100.0 | 17.5 ± 2.2 |  |  | COWAT |  |  |  |
|  |  |  |  |  |  |  |  | WCST | Mental flexibility | NA |  |
|  |  |  |  |  |  |  |  | ROCFT immediate copy | Visuospatial processing | *p* = 0.198 |  |
|  |  |  |  |  |  |  |  | Naming Test | Verbal fluency | NA |  |
|  |  |  |  |  |  |  |  | Animal Fluency | Verbal fluency | NA |  |
|  |  |  |  |  |  |  |  | Barratt Impulsivity Scale | Impulsivity | NS |  |
| Choi et al., 2018 | Cross-sectional comparative | 275 smell impairment control | 71.9 ± 7.1 | 65.8 | NA | USA | Pocket  Smell Test  Score (identification) | WAIS - Digit  Symbol Substitution | Executive function | *r* = 0.31, *p* < 0.001; *β* =10.2, *p* < 0.001 | Pearson’s correlation/linear regression  (*α* = 0.05) |
|  |  |  |  |  |  |  |  | Animal  Fluency Test | Verbal fluency | *r* = 0.31, *p* < 0.001; *β* = 2.90, *p* < 0.001 |  |
|  |  | 961 control | 68.5 ± 6.3 | 49.4 | NA |  |  |  |  |  |  |
| Herman et al., 2018 | Cross-sectional | 79 control | 22.1 ± 3.4 | 38.0 | NA | UK | Sniffin’ Sticks Test (detection, discrimination, and identification) | Stop-Signal Task | Inhibitory control | Detection: *β* = 4.50, *p* = 0.014; Discrimination *β* = -3.66, *p* = 0.026 | Multiple regression (covariates gender, age, and alcohol and cigarettes consumption; *α* = 0.05) |
|  |  |  |  |  |  |  |  | Barratt Impulsivity Scale (non-Planning) | Impulsivity | Discrimination *β* = -0.58, *p* = 0.012 |  |
|  |  |  |  |  |  |  |  | Information Sampling Task | Impulsivity | NS |  |
|  |  |  |  |  |  |  |  | Monetary Choice Questionnaire | Impulsivity | NS |  |
| Churnin et al., 2019 | Cross-sectional comparative | 312 olfactory dysfunction control | 72.3 ± 7.1 | 59.6 | NA | USA | Pocket  Smell Test  Score (identification) | Animal  Fluency Test ( AFT) | Verbal fluency | AFT (OR 2.88, *p* *<* 0.001) | Logistic regression  (*α* = 0.05) |
|  |  |  |  |  |  |  |  | Digit Symbol Substitution Test | Executive function | DSST (OR *=* 6.05, *p* *<* 0.001) |  |
|  |  | 1064 control | 68.7 ± 6.4 | 46.0 | NA |  |  |  |  |  |  |
| Freimer et al., 2019 | Cross-sectional | 20 OI tertile 1 | 81.2 ± 7.3 | 95 | 16.0 ± 2.1 | USA | OLFACT olfactometer (identification) | Executive composite (TMT and WAIS - Digit Symbol) | Executive function | Overall p = 0.05; OI Tertile 1 vs. OI Tertile 2, *p* = NS; OI Tertile 1 vs. OI Tertile 3, *p* = NS | multivariable linear regression, Tukey’s HSD post-hoc test |
|  |  | 20 OI tertile 2 | 81.7 ± 9.8 | 25 | 14.7 ± 2.1 |  |  |  |  |  |  |
|  |  |  |  |  |  |  |  |  |  |  |  |
|  |  | 19 OI tertile 3 | 73.8 ± 7.8 | 15.8 | 14.8 ± 2.3 |  |  |  |  |  |  |
| Yahiaoui et al., 2019 | Cross-sectional | 6783 control | NA | 49.0 | NA | Germany | Sniffin’ Sticks Test (detection, discrimination, and identification) | TMT B  Consortium to Establish a Registry for Alzheimer’s  Disease - verbal fluency | Mental flexibility  Verbal fluency | Smell test sore: B/A ratio *β* = -0.16, *p* *<* 0.001  Smell test sore: *β* = 0.79, *p* < 0.001 | Linear regression (*α* = 0.05) |
| Turana et al., 2020 | Cross sectional | 470 Indonesians  819 White Australians | 67.4 ± 7.4  78.7 ± 4.8 | 31.8%  43.2% | NA | Indonesia and Australia | 10-item test of odors commonly found in Indonesia  Brief Smell Identification Test | semantic fluency test (animals named in 60 s) | Verbal fluency | Indonesians: b = 0.121, p = 0.001; White Australians: b = 0.054, p = 0.002 | Univariate general linear model |
| Cha et al., 2021 | Cross-sectional | 108 eldery controls | 75.74 ± 6.63 | 49.1% | NA | South Korea | UPSIT | SNSB II  (Frontal/executive subset)  Visuospatial | Executive function  Visuospatial processing | *β* =0.092  t(*p*) = 1.036 (0.30)  *β* =0.559 (0.577)  t(*p*) = 0.559 (0.577) | ANCOVA (covariates:  type, and CDR score) |
| Gellrich et al., 2021 | Cross-sectional | 100 young controls | 8.5 ± 1.7 | 50% | NA | Germany | Sniffin' Sticks | NA | NA | NA | NA |
|  |  | 100 older controls | 14.5 ± 1.8 | 48% | NA |  |  | WCST | Mental flexibility | P = NS | Spearman correlations ( *α* = 0.05) |
| Kose et al., 2021 | Cross-sectional | 18 OI tertile 1 | 73.0 ± 6.2 | 28% | NA | Japan | Odor Stick Identification Test for Japanese (OSIT-J) | TMT B | Executive function | P = 0.33 (not adjusted for and gender), p = NS (adjusted for age and gender); | ANOVA, ANCOVA (adjusted for age and gender) |
|  |  | 13 OI tertile 2 | 74.4 ± 5.5 | 38% |  |  |  |  |  |  |  |
|  |  |  |  |  |  |  |  |  |  |  |  |
|  |  | 13 OI tertile 3 | 69.5 ± 4.0 | 31% |  |  |  |  |  |  |  |

Control group refers to individuals without the condition associated with olfactory dysfunction. Values represent mean ± standard deviation. NA – not available; NS – not statistically significant but results are not reported; AD – Alzheimer’s disease; AFT – Animal Fluency Test; ALS - Amyotrophic lateral sclerosis; aMCI – Amnestic mild cognitive impairment; BST-24- Barcelona Smell Test-24; BRI: behavioral regulation index; bvFTD – behavioral variant frontotemporal dementia; βs - standardized regression coefficient; β – regression coefficient; CAMCOG- Cambridge Cognition Examination;CBS - corticobasal syndrome; COWAT - Control Oral Word Association Test; CCSIT - Cross-Cultural Smell Identification Test; DKEFS - Delis–Kaplan Executive Function System; DSST- Digit Symbol Substitution Test; eoAD: early-onset Alzheimer’s disease; eoMCI- early-onset mild cognitive impairment; EF- Executive functioning ; VD - Vascular dementia; fvFTD - Frontal variant frontotemporal dementia; GEC: The global executive composite; and IED: Intermittent Explosive Disorder; MCI- Metacognition index; MoCA- Montreal Cognitive Assessment Test; MS – Multiple sclerosis; naMCI – nonamnestic mild cognitive impairment; NORM- normosmic; OSIT-J - Odor Stick Identification Test for Japanese; PD – Parkinson’s disease; PEA – Phenyl ethyl alcohol; PTSD – Post-Traumatic Stress Disorder; LLD- late life depression, RBANS- Repeatable Battery for the Assessment of Neuropsychological Status, r – correlation test value; LLD patients without OI impairment (LLD-NOII); SMI – Subjective memory impairment; SCWT - Stroop Color Word Test; SCD- subjective cognitive decline; SNSB II: The Seoul Neuropsychological Screening Battery II; TBI – Traumatic brain injury; TMT - Trail Making Test; OCD – Obsessive-compulsive disorder; OD – Olfactory dysfunction; OI – Olfactory identification; OR – odds ratio; ROCFT - Rey-Osterrieth Complex Figure Test; UPSIT- University of Pennsylvania Smell Identification Test ; VFT-verbal fluency test; WAIS - Wechsler Adult Intelligence Scale; WMS - Wechsler Memory Scale; WCST - Wisconsin Card Sorting Test.
