# Supplementary material for: Olfaction and Executive Cognitive Performance: A Systematic Review
Source: Front Psychol. 2022 May 9;13:871391. doi: 10.3389/fpsyg.2022.871391 (PMC9125097; doi:10.3389/fpsyg.2022.871391)
Supplement: Supplementary file 2 [file Table_2.docx]

| **Table 2** Quality assessment of the studies included in the systematic review based on the items adapted from the National Institutes for Health tool for observational cohort and cross-sectional studies. | | | | | | | | | | | | |
| --- | --- | --- | --- | --- | --- | --- | --- | --- | --- | --- | --- | --- |
| **Study** | **Adequate definition of research question/objective** | **Adequate definition of study population** | **Indication of ≥50% participation rate** | **Inclusion and exclusion criteria for all participants** | **Justification of sample size/ description of power and effect sizes** | **Adequate definition of exposure measures** | **Adequate definition of outcome measures** | **Blinding of outcome assessors** | **Indication of ≤20% exclusion rate** | **Assessment of confounding variables effects** | **Statistical analysis for all groups** | **Adequate reporting with information regarding statistical Methods** |
| Seidman et al., 1992 | Yes | Yes | Yes | Yes | No | Yes | Yes | NR | NR | Yes | Yes | Yes |
| Brewer et al., 1996 | Yes | Yes | Yes | Yes | No | Yes | Yes | NR | NR | Yes | Yes | Yes |
| Seidman et al., 1997 | Yes | Yes | Yes | Yes | No | Yes | Yes | Yes | Yes | No | Yes | Yes |
| Purdon, 1998 | Yes | Yes | Yes | Yes | No | Yes | Yes | Yes | Yes | No | Yes | Yes |
| Saoud et al., 1998 | Yes | Yes | Yes | Yes | No | Yes | Yes | NR | NR | Yes | Yes | Yes |
| Barnett et al.,1999 | Yes | Yes | Yes | Yes | No | Yes | Yes | NR | Yes | Yes | Yes | Yes |
| Callahan and Hinkebein,1999 | Yes | Yes | NR | NR | No | Yes | Yes | NR | NR | No | Yes | Yes |
| Vasterling et al., 2000 | Yes | Yes | NR | Yes | No | Yes | Yes | NR | NR | No | Yes | Yes |
| Spinella, 2002 | Yes | No | NR | NR | No | Yes | Yes | NR | NR | No | Yes | Yes |
| Vasterling et al., 2003 | Yes | Yes | Yes | Yes | Yes | Yes | Yes | NR | NR | No | Yes | Yes |
| Larsson et al., 2004 | Yes | Yes | Yes | Yes | No | Yes | Yes | NR | Yes | Yes | Yes | Yes |
| Corcoran et al., 2005 | Yes | Yes | Yes | Yes | No | Yes | Yes | NR | NR | Yes | Yes | Yes |
| Westervelt et al., 2005 | Yes | Yes | Yes | Yes | No | Yes | Yes | NR | Yes | Yes | Yes | Yes |
| Killgore and Mcbride, 2006 | Yes | Yes | Yes | Yes | No | Yes | Yes | NR | NR | Yes | NR | Yes |
| Rupp et al., 2006 | Yes | Yes | Yes | Yes | No | Yes | Yes | NR | Yes | Yes | Yes | Yes |
| Dileo et al., 2008 | Yes | Yes | Yes | Yes | No | Yes | Yes | NR | NR | Yes | Yes | Yes |
| Lehrner et al., 2009 | Yes | Yes | Yes | Yes | No | Yes | Yes | NR | NR | No | Yes | Yes |
| Pardini et al., 2009 | Yes | Yes | Yes | Yes | No | Yes | Yes | NR | NR | No | Yes | Yes |
| Bahar-Fuchs et al., 2010 | Yes | Yes | Yes | Yes | No | Yes | Yes | NR | Yes | Yes | Yes | Yes |
| Devanand et al., 2010 | Yes | Yes | Yes | Yes | No | Yes | Yes | NR | Yes | No | Yes | Yes |
| Hedner et al., 2010 | Yes | Yes | Yes | Yes | No | Yes | Yes | NR | Yes | Yes | Yes | Yes |
| Killgore et al., 2010 | Yes | Yes | Yes | Yes | No | Yes | Yes | Yes | NR | Yes | Yes | Yes |
| Sigurdardottir et al., 2010 | Yes | Yes | Yes | Yes | Yes | Yes | Yes | NR | Yes | Yes | Yes | Yes |
| Morley et al. 2011 | Yes | Yes | NR | NR | No | Yes | Yes | NR | Yes | Yes | Yes | Yes |
| Maurage et al., 2011 | Yes | Yes | NR | Yes | No | Yes | Yes | NR | NR | Yes | Yes | Yes |
| Segalas et al., 2011 | Yes | Yes | Yes | Yes | No | Yes | Yes | NR | NR | Yes | Yes | Yes |
| Tkalčić et al., 2011 | Yes | Yes | Yes | NR | No | Yes | Yes | NR | NR | Yes | Yes | Yes |
| Hardy et al., 2012 | Yes | Yes | Yes | NR | No | Yes | Yes | NR | Yes | No | Yes | Yes |
| Lee et al., 2012 | Yes | Yes | Yes | Yes | No | Yes | Yes | NR | NR | Yes | Yes | Yes |
| Parrao et al., 2012 | Yes | Yes | Yes | Yes | No | Yes | Yes | NR | NR | No | Yes | Yes |
| Bersani et al., 2013 | Yes | Yes | Yes | Yes | No | Yes | Yes | NR | NR | Yes | Yes | Yes |
| Bettison et al., 2013 | Yes | Yes | Yes | Yes | No | Yes | Yes | NR | Yes | Yes | Yes | Yes |
| Hanoglu et al., 2014 | Yes | Yes | Yes | Yes | No | Yes | Yes | NR | NR | No | Yes | Yes |
| Kjelvik et al., 2014 | Yes | Yes | Yes | Yes | No | Yes | Yes | NR | NR | No | Yes | Yes |
| de Guise et al., 2015 | Yes | Yes | Yes | Yes | Yes | Yes | Yes | NR | NR | No | Yes | Yes |
| Devanand et al., 2015 | Yes | Yes | Yes | Yes | No | Yes | Yes | NR | Yes | No | Yes | Yes |
| Fagundo et al., 2015 | Yes | Yes | Yes | Yes | No | Yes | Yes | NR | NR | Yes | Yes | Yes |
| Vyhnalek et al., 2015 | Yes | Yes | Yes | Yes | Yes | Yes | Yes | NR | NR | Yes | Yes | Yes |
| Crowley and McDonald, 2016 | Yes | Yes | Yes | NR | No | Yes | Yes | NR | NR | Yes | NR | Yes |
| Orasji et al., 2016 | Yes | Yes | Yes | Yes | No | Yes | Yes | NR | NR | No | Yes | Yes |
| Pilotto et al., 2016 | Yes | Yes | Yes | Yes | No | Yes | Yes | NR | Yes | Yes | Yes | Yes |
| Sigurdardottir et al., 2016 | Yes | Yes | Yes | Yes | No | Yes | Yes | NR | NR | Yes | Yes | Yes |
| Ward et al., 2016 | Yes | Yes | Yes | Yes | Yes | Yes | Yes | NR | Yes | Yes | Yes | Yes |
| Alosco et al., 2017 | Yes | Yes | Yes | Yes | No | Yes | Yes | NR | Yes | Yes | Yes | Yes |
| Bakker et al., 2017 | Yes | Yes | Yes | Yes | No | Yes | Yes | NR | Yes | Yes | Yes | Yes |
| Goette et al., 2017 | Yes | Yes | Yes | NR | No | Yes | Yes | NR | NR | Yes | Yes | Yes |
| Carotenuto et al., 2018 | Yes | Yes | Yes | Yes | No | Yes | Yes | NR | NR | Yes | Yes | Yes |
| Chen et al. 2018 | Yes | Yes | Yes | Yes | No | Yes | Yes | NR | NR | Yes | Yes | Yes |
| Choi et al., 2018 | Yes | Yes | Yes | Yes | No | Yes | Yes | NR | NR | Yes | Yes | Yes |
| Herman et al., 2018 | Yes | Yes | Yes | Yes | No | Yes | Yes | NR | Yes | Yes | Yes | Yes |
| Park et al., 2018 | Yes | Yes | Yes | Yes | No | Yes | Yes | Yes | NR | No | Yes | Yes |
| Takahashi et al., 2018 | Yes | Yes | Yes | Yes | No | Yes | Yes | NR | Yes | Yes | Yes | Yes |
| Blanco et al., 2019 | Yes | Yes | Yes | NR | No | Yes | Yes | NR | NR | Yes | Yes | Yes |
| Churnin et al. 2019 | Yes | Yes | Yes | Yes | No | Yes | Yes | NR | Yes | Yes | Yes | Yes |
| Freimer et al., 2019 | Yes | Yes | Yes | Yes | No | Yes | Yes | NR | NR | No | Yes | Yes |
| Giguere et al., 2019 | Yes | Yes | Yes | Yes | No | Yes | Yes | NR | NR | No | Yes | Yes |
| Lian et al., 2019 | Yes | Yes | Yes | Yes | No | Yes | Yes | NR | NR | Yes | Yes | Yes |
| Mertens et al., 2019 | Yes | No | Yes | NR | No | Yes | Yes | NR | NR | No | Yes | Yes |
| Velayodhan et al., 2019 | Yes | Yes | Yes | Yes | No | Yes | Yes | NR | NR | Yes | Yes | Yes |
| Yahiaoui et al., 2019 | Yes | Yes | Yes | Yes | No | Yes | Yes | NR | Yes | Yes | Yes | Yes |
| Yoshii et al., 2019 | Yes | Yes | Yes | Yes | No | Yes | Yes | NR | NR | No | Yes | Yes |
| Tahmasebi et al., 2020 | Yes | Yes | Yes | Yes | No | Yes | Yes | NR | NR | Yes | Yes | Yes |
| Turana et al., 2020 | Yes | Yes | Yes | Yes | No | Yes | Yes | NR | Yes | No | Yes | Yes |
| Yoo et al., 2020 | Yes | Yes | Yes | Yes | No | Yes | Yes | NR | NR | Yes | Yes | Yes |
| Cha et al., 2021 | Yes | Yes | Yes | Yes | Yes | Yes | Yes | NR | Yes | Yes | Yes | Yes |
| Gellrich et al., 2021 | Yes | Yes | Yes | NR | No | Yes | Yes | NR | Yes | No | Yes | Yes |
| Langdon et al., 2021 | Yes | Yes | Yes | Yes | No | Yes | Yes | NR | NR | Yes | Yes | Yes |
| Kose et al., 2021 | Yes | Yes | Yes | NR | No | Yes | Yes | NR | NR | Yes | Yes | Yes |
| Wang et al., 2021 | Yes | Yes | Yes | Yes | No | Yes | Yes | NR | NR | Yes | Yes | Yes |

NR – not reported
